# Synthesis, Crystal Structure, DFT Calculations, Hirshfeld Surface Analysis and In Silico Drug-Target Profiling of (*R*)-2-(2-(1,3-Dioxoisoindolin-2-yl)propanamido)benzoic Acid Methyl Ester

**DOI:** 10.3390/molecules28114375

**Published:** 2023-05-26

**Authors:** Syeda Laila Rubab, Abdul Rauf Raza, Bushra Nisar, Muhammad Ashfaq, Yasir Altaf, Riaz Hussain, Noreen Sajjad, Muhammad Safwan Akram, Muhammad Nawaz Tahir, Muhammad Ashraf Shaheen, Muhammad Fayyaz ur Rehman, Hayssam M. Ali

**Affiliations:** 1 Department of Chemistry, Division of Science and Technology, University of Education, Lahore 54770, Pakistan; 2 Institute of Chemistry, Ibn e Sena Block, University of Sargodha, Sargodha 40100, Pakistan; 3 Department of Chemistry, The University of Lahore, Sargodha Campus, Sargodha 40100, Pakistan; 4 Department of Physics, University of Sargodha, Sargodha 40100, Pakistan; 5 School of Chemical and Physical Sciences, Victoria University of Wellington, Wellington 6140, New Zealand; 6 Department of Chemistry, The University of Lahore, Lahore 54770, Pakistan; 7 School of Health and Life Sciences, Teesside University, Middlesbrough TS1 3BA, UK; 8 Department of Botany and Microbiology, College of Science, King Saud University, Riyadh 11451, Saudi Arabia

**Keywords:** isoindole, DFT, crystal structure, molecular docking, antimicrobials

## Abstract

The work here reflects synthesis, DFT studies, Hirshfeld charge analysis and crystal data exploration of pharmacologically important (*R*)-2-(2-(1,3-dioxoisoindolin-2-yl)propanamido)benzoic acid methyl ester (**5**) to understand its properties for further chemical transformations. The methyl anthranilate (**2**) was produced by the esterification of anthranilic acid in an acidic medium. The phthaloyl-protected alanine (**4**) was rendered by the fusion of alanine with phthalic anhydride at 150 °C, followed by coupling with (**2**) furnished isoindole (**5**). The characterization of products was performed using IR, UV-Vis, NMR and MS. Single-crystal XRD also verified the structure of (**5**) in which N-H⋯O bonding stabilizes the molecular configuration of (**5**), resulting in the formation of S(6) hydrogen-bonded loop. The molecules of isoindole (**5**) are connected in the form of dimers, and the π⋯π stacking interaction between aromatic rings further stabilizes the crystal packing. DFT studies suggest that HOMO is over the substituted aromatic ring, the LUMO is present mainly over the indole side, and nucleophilic and electrophilic corners point out the reactivity of the product (**5**). In vitro and in silico analysis of (**5**) shows its potential as an antibacterial agent targeting DNA gyrase and Dihydroorotase from *E. coli* and tyrosyl-tRNA synthetase and DNA gyrase from *Staphylococcus aureus*.

## 1. Introduction

Isoindoles belong to an important class of heterocyclic compounds of pharmaceutical importance. The substituted isoindoles have significant therapeutic potential and remain a long-standing focus of dynamic scientific interest. It is a large group of salutary agents showing anti-HIV [1], anticancer [2], antipsychotic [3], antianxiety [4,5], anti-inflammatory [6], antimicrobial [7,8], antiarrhythmic [9] and anticonvulsant [10] activities. The molecular modification and derivatization of available drugs have led to enormously improved medicines.

**Scheme 1:**
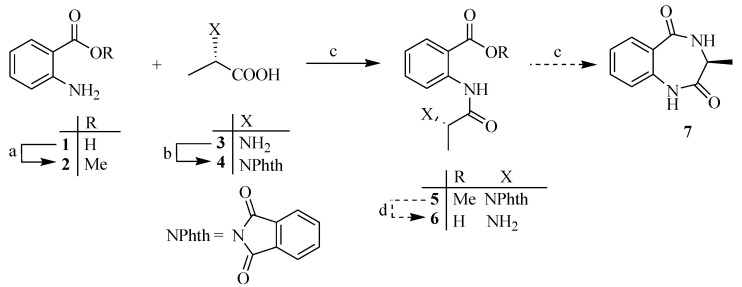
(**a**) MeOH, H_2_SO_4_ (cat.) at 60 °C (72 h); (**b**) phthalic anhydride at 155 °C (1 h); (**c**) SOCl_2_ at 50 °C (6 h); (**d**) aq. MeNH_2_, room temperature.

We have already successfully synthesized chiral seven-membered rings of benzoxazepines [11]. During our current studies, attempts were made to synthesize chiral 1,4-benzodiazepines. We have tried to develop a simple method of preparation of asymmetric benzodiazepines by coupling methyl ester of anthranilic acid with phthaloyl-protected (S)-alanine. The reaction yielded a crystalline product as an exclusive product with a significant yield. Spectroscopic characterization indicated that the desired product was not formed; instead, an isoindole (**5**) was formed, which failed to be further converted into benzodiazepine **7** (Scheme 1). The resulting substituted isoindole **5** is an attractive pharmacophore for different activities and is important in medicines and drug chemistry. Considering the structure of the isoindole with anthranilic acid moiety, isoindole moiety and amino acid residue, we decided to apply DFT and Hirshfeld studies on the synthesized product to have a deeper insight into the chemical behavior and reactivity of this interesting product to further derivatize it as a potential drug. Considering the pharmacological importance mentioned above, we also decided to check the pharmacological screening of the achieved isoindole.

## 2. Results and Discussions

Anthranilic acid was esterified to form compound **2** to protect its carboxy-terminal (C-terminal). Further, the coupling of ester **2** with phthalamide **4** formed an expected coupled product **5**. The deprotection (ester and phthaloyl moieties) followed by conversion of resulting carboxylic acid **6** to COCl_2_ and the intramolecular cyclization was expected to construct diazepine ring **7**, which was abandoned due to the conversion of **5** back into **4** instead of the desired benzodiazepine **7** (Scheme 1) under applied conditions for cyclization reaction. The substituted isoindole **5** was prepared during the proposed route adopted for synthesizing benzodiazepine **7**, which was characterized by using different spectroscopic and mass spectrometric techniques followed by DFT studies.

### 2.1. Characterization

**Scheme 2:**
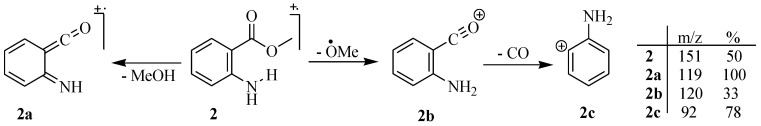
The EIMS fragmentation pattern of methyl anthranilate **2**.

The esterification of anthranilic acid employing acid catalysis formed **2** as purple-colored oil with a 50% yield by procedure mentioned in the literature [12]. The absence of the O–H signal and the presence of a broad C=O signal in the IR spectrum indicated successful esterification. Although the ^1^H-NMR spectrum of ester **2** showed an aromatic region similar to that of the reactant, three additional proton singlet peaks appeared, supporting the formation of methyl anthranilate **2**. The molecular ion peak of ester appeared at 151 amu, having a 14 amu increase on the molecular ion peak of anthranilic acid, confirming successful esterification. The MeO radical and CO were lost successively, supporting a logical fragmentation pattern. The rearranged fragment (β-keto acid) generated via the MeOH removal from [M^+•^] is attributed as the base peak (119 amu) (Scheme 2) (Supplementary Materials; Compound characterization data).

It was essential to protect the NH_2_ terminal of (*S*)-alanine **2** to avoid interference during the coupling reaction. The *N*-protection of (*S*)-alanine **3** was carried out by the fusion of amino acid with phthalic anhydride [13]. The removal of unreacted phthalic anhydride was easy after the completion of the reaction due to sublimation. The reaction is highly attractive for being environmentally friendly and efficient with a high yield (70–80%). This condensation resulted in the formation of crystalline **4** with significant yields (Table 1).

**Scheme 3:**
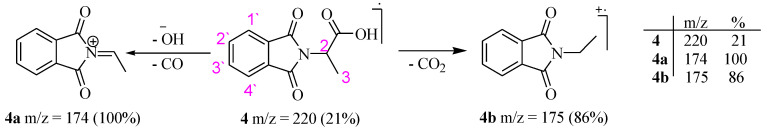
The EIMS fragmentation pattern of *N*-protected alanine **4**.

Compound **4** exhibited molecular ion peaks that were in accordance with the expected cyclized product for successful *N*-protection. The loss of OH radical and CO or CO_2_ gave daughter fragments, confirming the formation of the required product (Scheme 3).

The signals of four aromatic region protons and the presence of peaks in the aliphatic region of ^1^H-NMR also suggested successful *N*-protection. In product **4,** H^2^ appeared at 7.80–7.90 ppm as a multiplet (Table 2).

**Figure 1:**
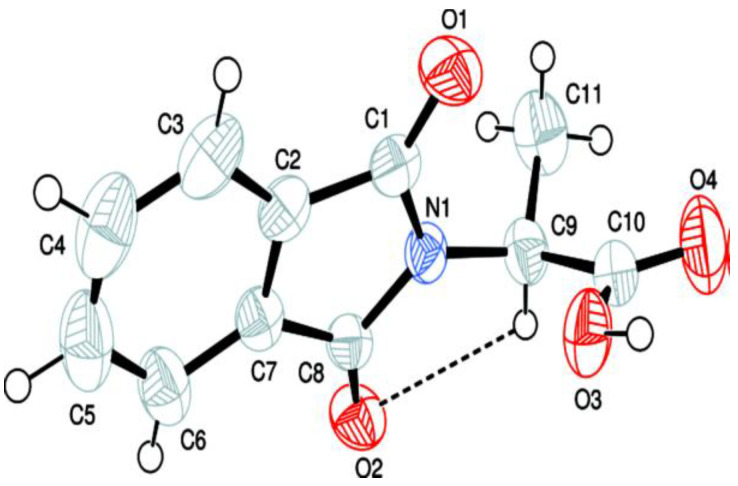
The ORTEP presentation of *N*-protected alanine **4**.

Concrete evidence for *N*-protection of **3** was shown by single-crystal XRD (Figure 1).

The acid chloride of *N*-protected alanine **4** was synthesized by refluxing it (1 h) with SOCl_2_. This acid chloride was then coupled with ester **2** by heating for 4 h at 40 °C, yielding colorless crystals of isoindole **5**.

The IR spectrum of the isoindole **5** showed distinctive absorbance analogous to the three C=O groups at 1770, 1707, 1595 cm**^−^**^1^ and one N–H at 3269 cm^−^^1^. The ^1^H-NMR identified eight aromatic protons. Phthaloyl 4H, in the same electronic environment, appeared as two multiplets, each with 2H. The aliphatic proton signals also agreed with the expected values, indicating a doublet of three CH_3_ protons, a quartet of H^1΄^ and a singlet relative to 3H of OMe. The anthranilate portion showed the presence of four aromatic protons. The splitting pattern of this portion was similar to that of methyl anthranilate **2**; however, all these protons were relatively deshielded post-coupling, such as the H^3″^ signal that jumped most downfield from 6.75 ppm to 8.70 ppm.

**Figure 2:**
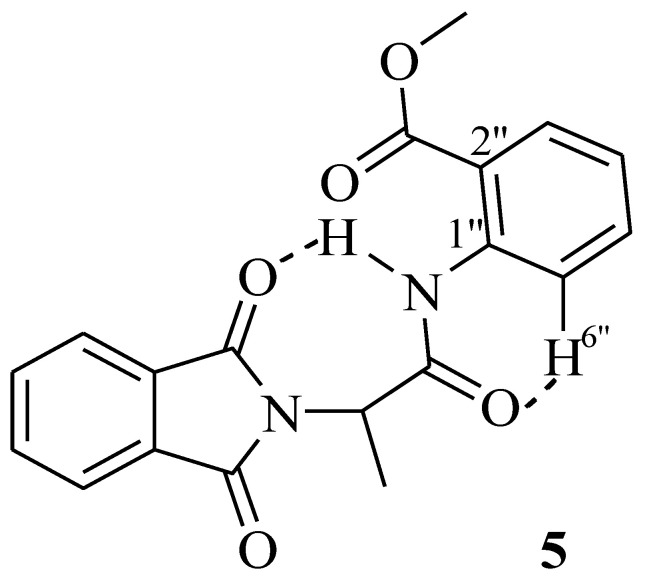
The electronic environment within the isoindole **5**.

The low field shift is justified due to the functional group interconversion of amino to the amido group; the amino group has +R while the amido group shows the −R effect. The field effect that deshields the proton H^3″^ is also a reason for the ppm shift (Figure 2).

The ^13^C-NMR analysis also supported the coupled product structure. The four C=O (CO_2_Me, C^3^, C^1^ and C^2′^) appeared at 172.0, 168.8, 168.0 and 167.9 ppm, respectively, whereas eight CHs of the aromatic region and four q Cs appeared in the range 120.5–134.2 ppm. The OCH_3_ carbons and CH_3_ are visible at 52.3, 50.1 and 15.1 ppm, respectively (Supplementary Materials; Compound characterization data).

**Scheme 4:**
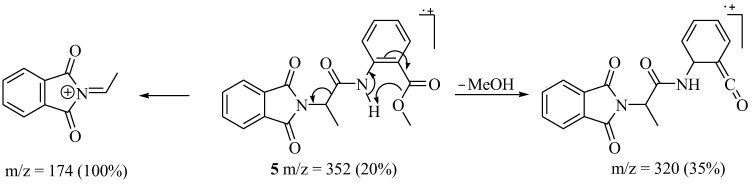
EIMS fragmentation pattern of isoindole **5**.

The high-resolution mass spectrum ultimately showed a peak of [M + Na]^+^ at 375.0943 amu, which agrees with the targeted product’s calculated mass. In the LR EIMS, the [M]^+•^ appeared at 352 amu. The further fragmentation mode and base peak also strengthened the proof for production of **5**. The MeOH removal gave a daughter fragment at 320 amu (Scheme 4).

Further reduction of **5** to yield **6** was a complete failure, and further conversion into **7** could not be carried out.

### 2.2. DFT Exploration

**Figure 3:**
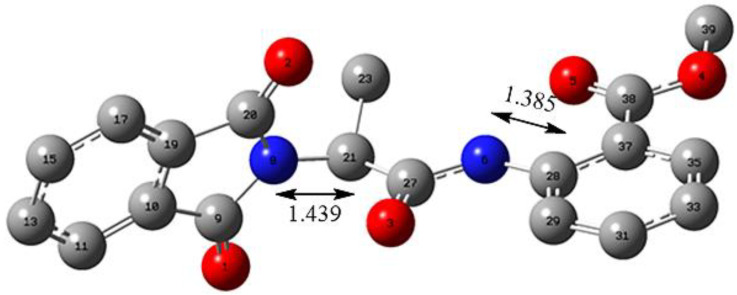
Optimized geometry compound (**5**) at PBE0-D3BJ/def2-TZVP level of theory. The grey color shows carbon in 3D models; red is for oxygen, and blue shows nitrogen atoms. Hydrogen atoms are omitted for clarity.

The three-dimensional structure of compound (**5**)**,** as shown in Figure 3 was modeled using Gauss View, and the geometry was then optimized at the PBE0-D3BJ/def2-TZVP level of density functional theory. The structure of compound (**5**) is a true minimum, as indicated by the absence of any imaginary frequency. Comparing the calculated structure with the crystallographic information shows that both results correlate well. For instance, the N8-C21 bond length is 1.460 Å, while N6-C28 is 1.396 Å in the crystal structure. The calculated bond lengths are 1.439 Å and 1.385 Å, with negligible deviation from the experimental bond lengths (Figure 3).

#### 2.2.1. Frontier Molecular Orbital Analysis (FMO) and Polarizability

**Figure 4:**
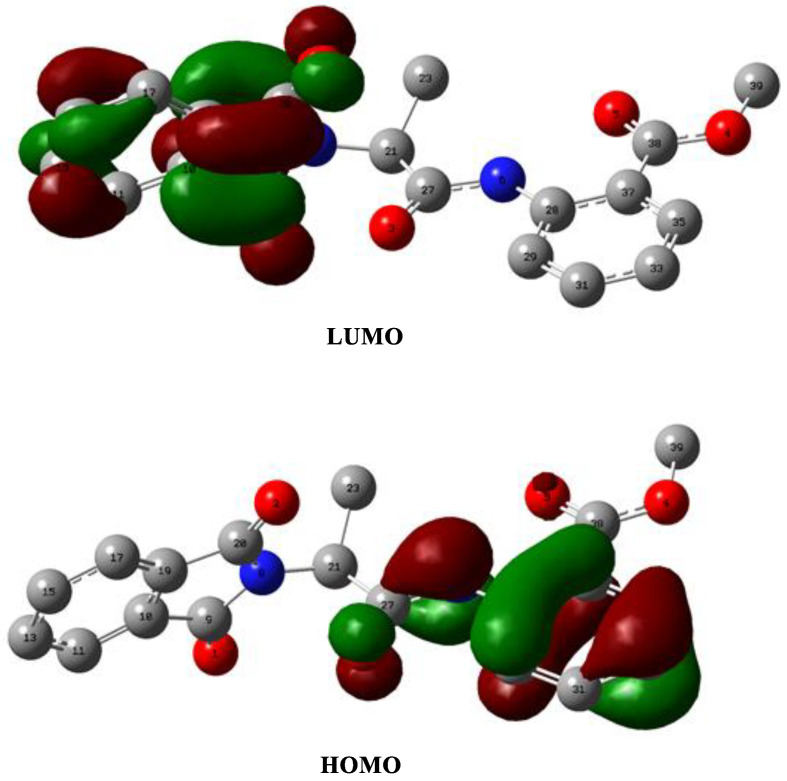
Isosurface plots of frontier molecular orbitals of the compound (**5**) calculated at PBE0-D3BJ/def2-TZVP level of theory. Hydrogen atoms are omitted for clarity.

FMO is one of the important tools for analyzing reactivity and describing many other physical parameters, including the energies of the highest occupied molecular orbital (HOMO) and the lowest unoccupied molecular orbitals (LUMO) [14]. The calculated HOMO-LUMO energy difference in the present case was 4.59 eV. Figure 4 shows the isosurface plots of HOMO and LUMO of the compound (**5**). It can be seen that HOMO is localized over the substituted aromatic ring (right side), and the LUMO exists mainly over the indole side. This further suggests that the nucleophilic side of the molecule is the right side (according to Figure 4), while the indole side is electrophilic. The hyperpolarizability (*β*) of compound (**1**) is 223.05 a.u., which shows that the molecule has a moderate non-linear optical response (Figure 4).

#### 2.2.2. Hirshfeld Charge Analysis

Hirshfeld charge analysis provides an insight into the spatial distribution of electron density in space throughout the molecule. This aids in understanding the intermolecular interactions within crystal packing [15]. Table 3 shows Hirshfeld charges of a few key atoms of the compound (**5**). It may be deduced that the oxygen (O1, O2, O3) and nitrogen (N8) atoms on the isoindole side are comparatively more negatively charged than the atoms on the other side. This, in turn, causes a positive charge on the adjacent carbon (C9, C20) atoms in the former case than those in the latter. However, C38 on the substituted aromatic ringside is the most positively charged due to the attachment of two oxygen atoms (Table 3).

### 2.3. Crystallographic Data

**Figure 5:**
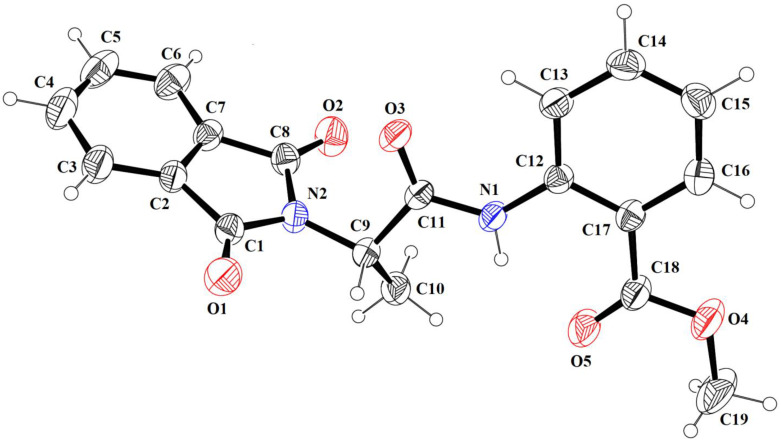
The ORTEP representation of (**5**) with a 30% probability level. H-atoms are expressed by tiny circles with random radii.

The ultimate evidence for coupled product **5** came from the single-crystal XRD structure. In **5** (Figure 5, Table 4) (CCDC no. 2059037), the isoindoline-1,3-dione group A (C1–C8/N2/O1/O2), acetamido moiety B (C9/C11/N1/O3) and methyl benzoate group C (C12–C19/O4/O5) are found planar having RMS (respective root mean square) deviation of 0.0331, 0.0083 and 0.0305 Å. The dihedral angle of acetamido moiety B with regard to groups A and C is 75.41 (**5**)° and 5.33 (**2**)°, respectively.

The ring comprising groups A and C has a dihedral angle of 73.27 (**3**)°. The C-atom (C10) is at the distance of 0.8382 (**3**) Å from the plane of moiety B. The important bond angles and bond lengths are specified in Table 5.

**Figure 6:**
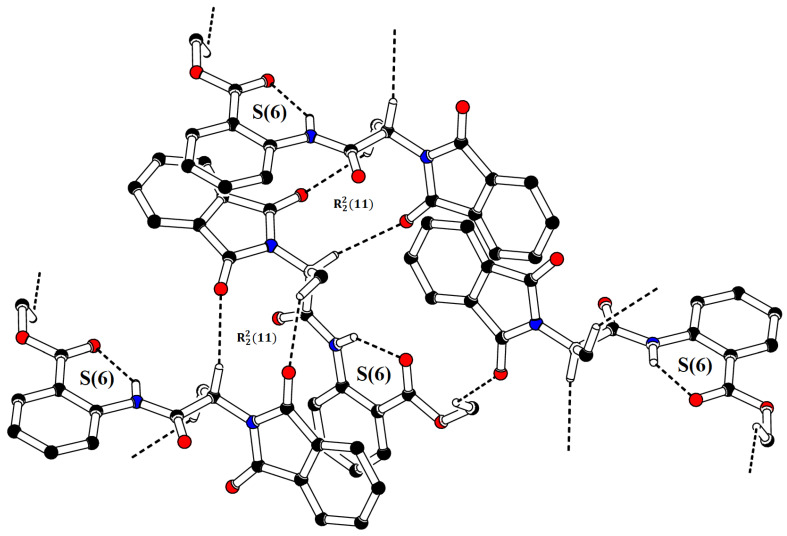
Packing representation of (**5**) (only specific H-atoms are expressed for clarity).

The molecular configuration is stabilized by intramolecular N-H⋯O bonding to form S (**6**) loop (Figure 6, Table 6). The molecules are connected with each other in the form of dimers through C-H⋯O bonding to form $R_{2}^{2}$(**11**) loops, where O-atoms of group A act as H-bond acceptors. The O-atom of moiety B is not involved in any intermolecular H-bonding. Due to above-mentioned C-H⋯O bonding, infinite chain is formed that runs along *b*-axis. CH of the methoxy group and O-atom of the carbonyl group (C1-O1) are engaged in C-H⋯O bonding.

**Symmetry codes:** (i) −*x* + 3/2, *y* + 1/2, −*z* + 1/2; (ii) −*x* + 3/2, *y* − 1/2, −*z* + 1/2; (iii) *x* + 1/2, − *y* + 3/2, *z* − 1/2.

**Figure 7:**
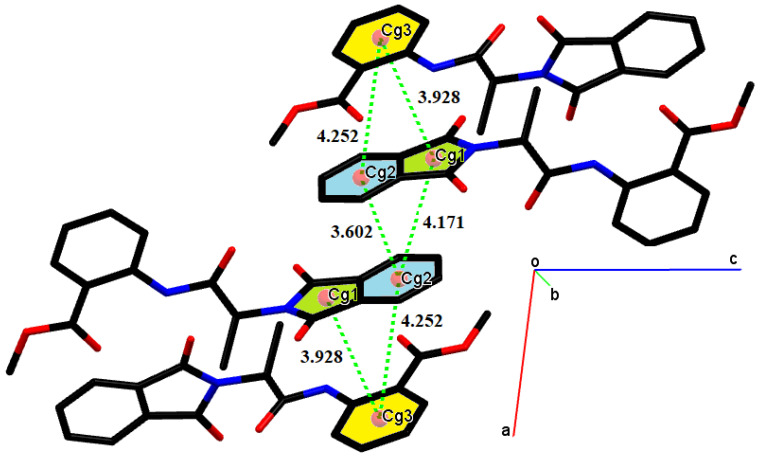
Graphical presentations of offset π⋯π interaction for (**5**) (hydrogen atoms are not shown to avoid ambiguity, and Å is used for distance calculation).

In addition to H-bonding, the crystal packing is further stabilized by offset π⋯π stacking interaction as shown in Figure 7. A strong offset stacking π⋯π interaction is formed between the phenyl ring Cg2 (C2–C7) of a molecule located in an asymmetric location and the phenyl ring Cg2 (C2–C7) located at (1-x, 1-y, 1-z) with an inter-centroid distance of 3.928 Å and ring offset of 0.424 Å, where Cg is the ring centroid. In general, the offset π⋯π stacking interaction is found between aromatic rings with inter-centroid distances ranging from 3.928 to 4.252 Å. The ring offset ranges from 0.776 to 2.060 Å.

**Figure 8:**
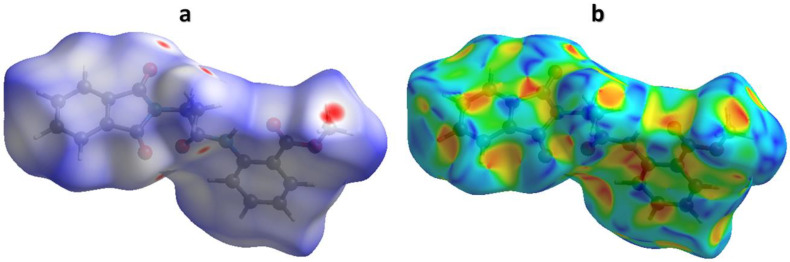
Hirshfeld surface plotted over (**a**) *d*_norm_ ranging from −0.1249 to 1.6990 a.u.; (**b**) shape index ranging from −1 to 1 a.u.

In single crystals, the crystal packing is stabilized by the non-covalent interactions. For the exploration of these interactions, the Hirshfeld surface (HS) is performed on Crystal Explorer, version 17.5 [16]. Color coding shows the non-covalent interactions on a Hirshfeld surface displayed on *d*_norm_ [17]. Red spots on HS indicate contacts for which the distance between atoms is smaller than the sum of the Van der Waal radii. White and blue regions represent weak and negligibly small interactions, respectively, as shown in Figure 8a. No red spot was found over the HS near the imine NH group, indicating the absence of N-H⋯N and N-H⋯O types of intermolecular H-bonding. Red spots on the HS are found near carbonyl O-atoms (O1/O2) of isoindoline-1,3-dione group A, CH of methoxy group, CH of carbon directly attached with group A and CH of the methyl group (C10), indicating that these atoms are engaged in H-bonding. Hirshfeld surface analysis can be used to explore the π⋯π stacking interaction between aromatic rings. For this, HS is plotted over the shape index. On this HS, the aromatic rings are seen to be involved in π⋯π stacking interaction due to the existence of consecutive triangular regions of red and blue colour (Figure 8b).

**Figure 9:**
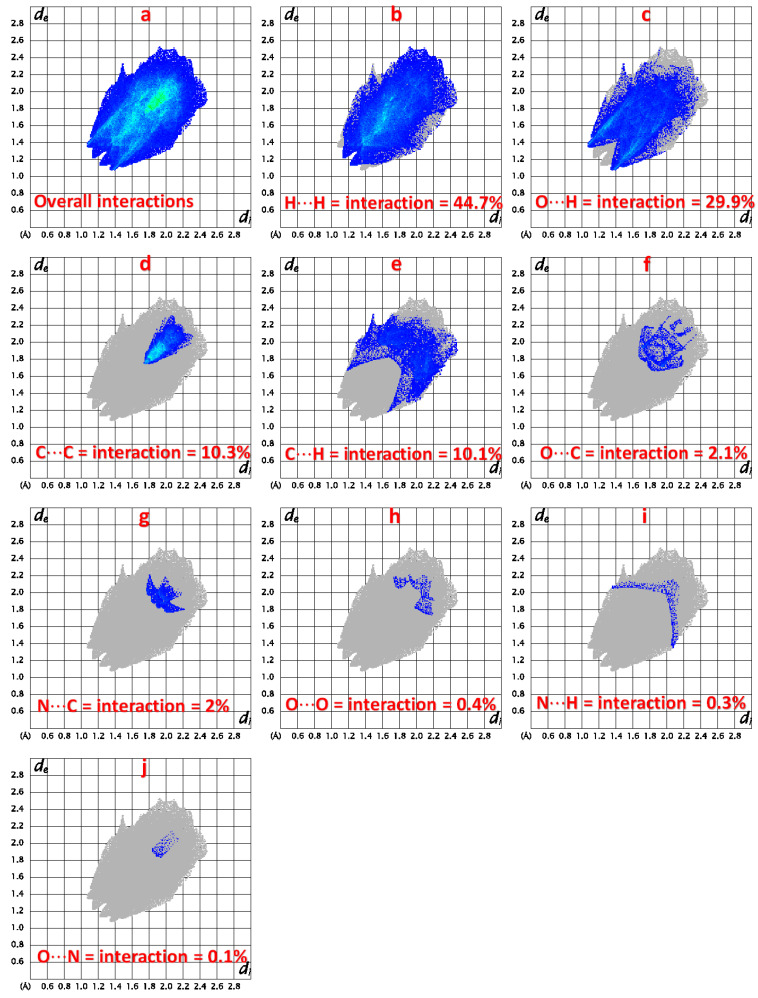
2D plots: (**a**) overall interactions and (**b**–**j**) individual interatomic contacts, including reciprocal contacts.

Intermolecular interactions in terms of individual interatomic contacts can be inspected by plotting 2D plots in which d_e_ represents the separation of HS to the closest nucleus outside the HS, whereas this distance from HS to the closest nucleus inside HS is represented by d_i_. Figure 9a shows a 2D plot for overall interactions. The central sky-blue zones of nearly triangular shape show that π⋯π stacking interaction is present in the crystal packing. Each interatomic contact is determined by considering its reciprocal contact. The interatomic contacts of type H⋯H, O⋯H, C⋯C and C⋯H are found to be the significant participants in crystal packing with %age contribution of 44.7% (Figure 9b), 29.9% (Figure 9c), 10.3% (Figure 9d) and 10.1% (Figure 9e), respectively. The interatomic contacts that have minor contribution to crystal packing are O⋯C (2.1%, Figure 9f), N⋯C (2%, Figure 9g), O⋯O (0.4%, Figure 9h), N⋯H (0.3%, Figure 9i) and O⋯N (0.1%, Figure 9j).

**Figure 10:**
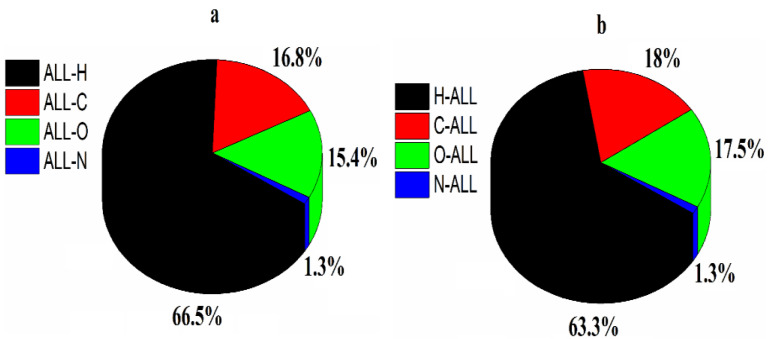
(**a**) Percentage contribution of the interaction of all the atoms found within HS to the atom located in surrounding HS; (**b**) percentage contribution of the interaction of an atom inside HS to all the atoms in the surrounding HS.

To learn more about crystal packing, computations were performed to determine how all the atoms present inside the HS interacts with the atom present outside the HS. Such types of interactions are quantized, as shown in Figure 10a, in which ALL represent all the atoms present inside HS. ALL-H interaction is the strongest interface, with a percentage impact of 66.5%. Other such interactions are ALL-N, ALL-O and ALL-C, with respective percentage contributions of 1.3%, 15.4% and 16.8% (Figure 10a). Likewise, the interaction of an atom within HS with atoms of molecules in its surroundings was investigated. In such interactions, ALL represents all the atoms of molecules present in the HS surrounding. H-ALL interaction is found to be the strongest interaction, with a percentage impact of 63.3%. Other such interactions are N-ALL, O-ALL and C-ALL, with respective percentage impacts of 1.3%, 17.5% and 18% (Figure 10b).

**Figure 11:**
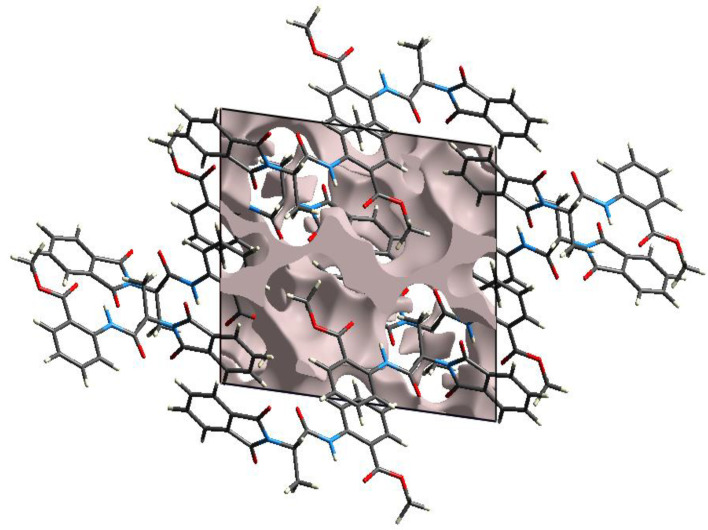
Graphical representation of voids in crystal packing.

In the unit cell, the percentage space occupied by the molecules define the crystal packing strength. If most of the space in the unit cell of a single crystal is occupied by voids, then it means crystal packing is weak. The void analysis was performed by assuming that the electron density around every atom is spherical. Graphical view of voids in compound **5** is shown in Figure 11. The volume of voids in the unit cell was 254.51 Å. In the unit cell, 14.6% of space was occupied by voids; in other words, 85.4% was occupied by atoms indicating that crystal packing is strong and has no large cavity.

### 2.4. In Vitro and In Silico Antimicrobial Activities

**Figure 12:**
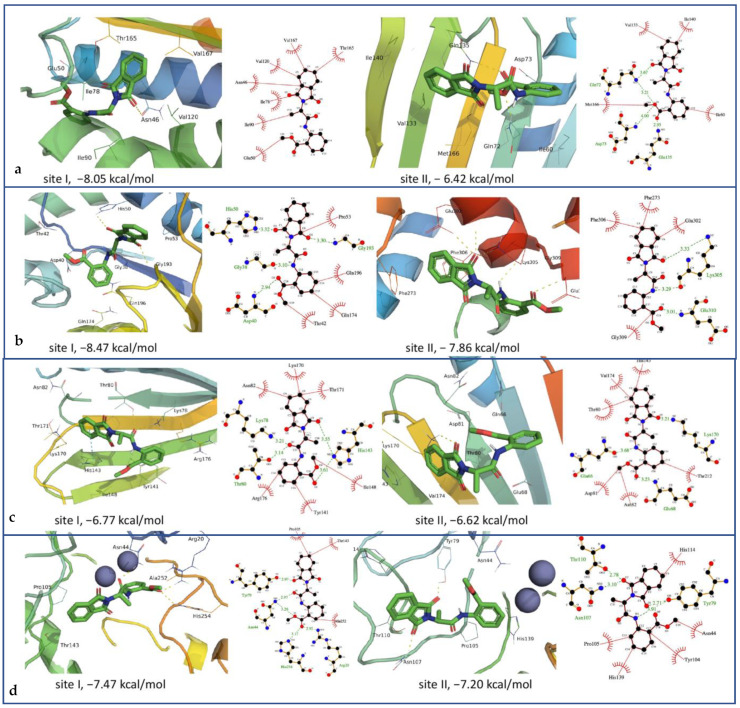
The ligand (**5**) interactions with Dihydroorotase from *E. coli* (**a**) tyrosyl-tRNA synthetase (**b**) and DNA gyrase from *S. aureus* (**c**) and *E. coli* (**d**). The ligands’ interactions are shown in ribbons and sheet diagrams, while interaction diagrams show lengths and number of H-bonds among (**5**) and enzyme active sites.

Disc diffusion assay revealed that (**5**) offers considerable bacterial activities against *E. coli* and *S. aureus.* The largest inhibition zone (16.50 mm) was observed against *S. aureus*, while an inhibition zone of 12.50 mm was observed against *E. coli*. In silico studies showed that (**5**) binds to multiple binding sites of *E. coli* DNA gyrase and Dihydroorotase, and to tyrosyl-tRNA synthetase and DNA gyrase from *Staphylococcus aureus* (Figure 12a–d). In the case of *E. coli* DNA gyrase, (**5**) binds to active site residues with −8.05 kcal/mol binding energy and 1.25 µM dissociation constant (Figure 12a, Table 7). The active site residues consisted of VAL43, ASN46, ASP49, ALA47, GLU50, VAL71, ILE90, ASP73, ARG76, MET91, GLY77, ILE78, VAL120, PRO79, ARG136, VAL167 and THR165 (Table 7). While binding to Dihydroorotase from *E. coli*, (**5**) interacted with −7.47 kcal/mol binding energy and shows a 3.40 µM dissociation constant (Table 7). The ligand also interacted with zinc metal ions in the enzyme active site. The binding site consisted of HIS18, ARG20, TYR79, ASN44, TYR104, PRO105, THR110, ASN107, HIS139, THR143, LEU222, HIS177, ASP250, HIS254, LA252 and ALA266 (Table 7). In another study, *N*-(benzo[*d*]thiazol-2-ylcarbamothioyl)-2/4-substituted benzamide had −7.4 kcal/mol binding energy against *E. coli* dihydroorotase. In Figure 12a,d, two different binding sites of (**5**) are shown against *E. coli* dihydroorotase and DNA gyrase. The dihydroorotase dehydrogenase enzyme is involved in de novo pyrimidine biosynthesis, while DNA gyrase is a topoisomerase involved in ATP-dependent negative super-coiling. Inhibition of either enzyme can affect the viability of *E. coli*. That is why these enzymes were screened as potential targets for the compound (**5**) to predict its mechanism of action as an antibacterial agent.

Against tyrosyl-tRNA synthetase of *S. aureus,* compound (**5**) showed very promising binding, with −8.47 KJ/mol binding energy and with 0.615 µM dissociation constant (Figure 12b, Table 7, while in the interaction with DNA gyrase (*S. aureus*), the binding energy and dissociation constant were −6.77 kcal/mol and 10.79 µM, respectively (Figure 12c). The tyrosyl-tRNA synthetase has binding sites that consist of GLY38, ALA39, THR42, ASP40, GLY49, HIS50, PHE54, PRO53, ASP80, GLY79, LYS84, ARG88, TYR170, GLY192, GLN174, GLY193, SER194, GLN196, ASP195, ASN199, LEU223, PRO222 and VAL224 (Table 7), and against *S. aureus* DNA gyrase binding site was consisted of LYS78, THR80, ASP81, ASN82, TYR141, HIS143, ILE148, LYS170, THR171, GLY172, VAL174 and ARG176 (Table 7). Previously, Triazinane and oxadiazinane molecular docking showed −9.37 kcal/mol, the highest binding energy against DNA gyrase. The in silico and in vitro activities of compound (**5**) seem to agree. The Dihydroorotase enzyme from *E. coli* and Tyrosyl-tRNA synthetase from *S. aureus* are vital enzymes, and these are necessary for the viability of these bacteria. Inhibiting these enzymes affects the growth and survival of bacteria in a growth media. The tyrosyl-tRNA synthetase is involved in aminoacylation; failing that leads to impaired protein synthesis and cellular functions.

## 3. Experimental Section

### 3.1. General Experimental Procedure

Freshly distilled analytical-grade organic solvents were used. The EtOAc, CHCl_3_, MeOH and EtOH were obtained from commercial sources. For crystallization and chromatography, a fraction of distilled hexane at 60*–*80 °C was employed. All solid chemicals were used as such.

Gallenkamp was used to record melting points (England, Serial MFB 595 010M) and reported uncorrected. For UV spectra, a Thermo Spectronic (UV-1700) spectrometer was used, and IR spectra were taken on IR Prestige 21 FTIR spectrophotometer, Shimadzu (Japan) at High Tech Laboratory, UoS, Sargodha. Molar absorptivity ε and absorption maxima are both reported. Equation *ε* = A/cl, was used to find *ε* values.

LR EIMS (low-resolution electron impact mass spectra) were recorded on Fisons Instrument VG Autospec mass spectrometer at ICCBS (International Center for Chemical and Biological Sciences), HEJRIC, University of Karachi, Karachi, while electrospray ionization mass spectra (ESI MS) were recorded using Q-TOF Ultima API (Micromass) at BMSF (Biomedical Mass Spectrometry Facility), UNSW (University of New Southwales), Sydney, Australia.

The NMR spectra of our products were recorded at ICCBS, HEJRIC, University of Karachi, Karachi, Pakistan, and at the School of Chemistry, University of New South Wales (UNSW), Sydney, Australia. The ^1^H NMR spectral data are shown as follows: chemical shifts in ppm downfield with reference to TMS (*δ*); proton number/count; multiplicity due to neighboring protons; observed J (coupling constant) in Hertz (Hz). Multiplicities are recorded in the form of s (singlet), bs (broad singlet), d (doublet), t (triplet), q (quartet), p (quintet), m (multiplet), dd (doublet of doublet), dt (doublet of triplet) and their combinations. Chemical shifts δ in the ^13^C NMR are reported downfield from TMS in ppm and show all identifiable carbons.

For recording XRD data to elucidate the structure of crystals, Bruker Kappa APEX 11 CCD diffractometer at the Department of Physics, UoS, was used. For the radiation source, fine-focus sealed tube monochromator graphite (Mo Kα radiation, λ = 0.71073 Å) was used.

We employed density functional tools under the realm of computational studies using the software Gaussian 09 [18]. We used PBE0 hybrid functional [19] and triple *ζ* basis set def2-TZVP [20] for our calculations. Dispersion interactions were included during optimization by adding Grimme’s empirical dispersion correction with Becke-Johnston damping (D3BJ) [17,21,22]. Calculations for frequency were performed to confirm the structure as a minimum on the potential energy surface.

### 3.2. Synthesis

#### 3.2.1. Methyl 2-Aminobenzoate (**2**)

The anthranilic acid **1** (1.00 g, 7.3 mmol, 1 eq) was dissolved in MeOH (7 mL), and 5.1 mL conc. H_2_SO_4_ (9.3 g, 0.095 mol, 13 eq) was added. The resulting solution was refluxed for 72 h while the reaction was monitored by TLC to ensure the completion of the reaction. A 1N aq. NaHCO_3_ solution was used to neutralize the solution and was extracted with n-hexane (2 × 20 mL). The n-hexane layer was filtered, dried over anhydrous Na_2_SO_4_ and rotary concentrated to obtain a purple-colored ester **2** (50%) in the liquid phase. R_f_; 0.6 (n-hexane: EtOAc, 1:1); density, 1.17 g/cm^3^; B.P. 256 ºC; log ε (λ_max_ in nm): 6.15 (337); ύ_max_ in cm*^−^*^1^, 1670 (C=O of conjugated ester), 3260 (N–H); δ_H_ in ppm (300 MHz): 7.79 (H^6^, dd of 1H, J =1.5, 8.4 Hz), 7.23 (H^4^, ddd of 1H, J = 1.5, 6.9, 8.4 Hz), 6.75 (H^3^, dd of 1H, J = 0.3, 8.1 Hz), 6.57 (H^5^, t of 1H, J = 7.8 Hz), 3.80 (s of 3H, OMe); LR EIMS (*m*/*z*, amu): 151 (50%) [M]^+•^, 120 (33%) [M–OCH_3_]^+^ A, 119 (100%) [M–CH_3_OH]^+•^, 92(78%) [A–CO]^+•^.

#### 3.2.2. (*2S*)-2-(1′,3′-Dioxoisoindolin-2-yl)propanoic Acid (**4**)

The (*S*)-alanine **3** (1 g, 0.02 mol, 1 eq) was fused with phthalic anhydride (0.03 mol, 1.1 eq) at about 155 °C for 60 min. The reaction completion was confirmed by the disappearance of reactants on TLC. The ninhydrin dip confirmed the absence of unreacted amino acids. The reaction flask was cooled to ambient temperature, and the unreacted crystallized phthalic anhydride was scratched off from the flask wall. The rest of the solid product was recrystallized from EtOH, and H_2_O yielded colourless prisms such as crystals of phthalamide **4** (750).

R_f_ = 0.26 (EtOOCH_3_); [α]^29^_D_ = –32.0 (c 1.5, EtOOCH_3_) M.P. = 160 °C; log ε (λ_max_ in nm) = 3.2003 (295.5); ύ_max_ in cm*^−^*^1^ KBr: 1708 (amide C=O), 1757 (carboxylic C=O), 3230 (O–H); δ_H_ in ppm (400 MHz): 7.80–7.90 (Ph, m of 4H), 5.00 (H^2^, m of 1H), 1.70 (Me, d of 3H, J = 7.2 Hz) LR EIMS (*m*/*z*, amu): 219 (21%) [M]^+•^, 175 (86%) [M^+•^–CO_2_], 174 (100%) [M^+•^*–*OH–CO].

#### 3.2.3. (R)-2-(2-(1,3-Dioxoisoindolin-2-yl)propanamido)benzoic Acid Methyl Ester (**5**)

The SOCl_2_ (0.7 mL, 7 eq) was added to phthalamide **4** (300 mg, 0.85 mmol, 1 eq) and heated at 100 °C until a clear solution was obtained. The addition of ester **2** (1 eq, 0.18 mL) was carried out to the reaction solution and was further heated for 5 h at 50 °C. The cooled solution was partitioned between H_2_O and CHCl_3_ (3×15 mL). The organic layer was filtered, dried over anhydrous Na_2_SO_4_ and concentrated under reduced pressure, which resulted in a pale, yellow-colored solid. It was crystallized from C_2_H_5_OH to form white-colored crystals (40%). R_f_: 0.5 (n-hexane:C_2_H_5_OAc, 1:1); M.P. 157 °C; log ε (λ_max_ in nm): 3.83024 (305); ύ_max_ in cm*^−^*^1^: 3269 for N–H, 1770 for ester C=O, 1707 for lactam C=O, 1595 for amide C=O; δ_H_ in ppm (300 MHz): 11.61 (NH, 1H), 8.70 (H^3″^, ddd of 1H, J = 8.7, 1.2, 0.6 Hz), 7.98 (H^6″^, ddd of 1H, J = 7.8, 1.8, 0.3 Hz), 7.08 (H^4″^, ddd of 1H, J = 1.2, 7.5, 8.7 Hz), 7.88–7.91 (H^4^, H^7^, m of 2H), 7.73–7.76 (H^5^, H^6^, m of 2H), 7.53 (H^5″^, dddd of 1H, J = 9.0, 7.2, 1.5, 0.3 Hz), 5.14 (H^1′^, q of 1H, J = 7.2 Hz), 3.72 (OMe, s of 3H), 1.87 (Me, d of 3H, J = 7.5 Hz); δ_C_ in ppm (75 MHz) 168.8 (ester C=O), 2 × s, 168.0 (C^1^ & C^3^); s, 167.9 (C^2′^); s, 141.2 (C^1″^); 2 × s, 134.9 (C^4a^ & C^7a^); 2 × d, 134.2 (C^4^ & C^7^); d, 132.3 (C^3″^); 2 × d, 131.0 (C^5^ & C^6^); d, 130.9 (C^5″^); d,123.0 (C^6″^); d, 120.5 (C^4″^); s, 115.3 (C^2″^); q, 52.3 (OMe); d, 50.1 (C^1′^); q 15.1(Me); LR EIMS (*m*/*z* in amu): 352 (20%) [M]^+•^, 320 (35%) [M*–*MeOH]^+*•*^, HR ESIMS [M + Na]^+•^ (*m*/*z* in amu) for C_19_H_16_N_2_O_5_ = 375.0997 (calc.), 375.0943 (observed), 5.4 mmu variation.

### 3.3. In Vitro and In Silico Analysis

Disk diffusion assay was performed as previously described [23]. Clinical isolates of *E. coli* and *S. aureus* were used to assess antibacterial activities, while Ciprofloxacin (1 mg/mL) was used as a standard drug. In silico studies were performed as described previously by our group. The protein structures were downloaded from the Protein Data Bank (PDB, https://www.rcsb.org/, accessed on 12 December 2022), and YASARA Structure ver. 22.10 was used for molecular docking with parameters used before [24]. The ligand-protein docking was visualized with PyMol ver. 2.5.4 [25] and LigPlot+ ver. 2.2.5 [26].

## 4. Conclusions

The structure of isoindole **5** is verified by the SC-XRD technique. The molecular configuration of isoindole **5** is stabilized due to N-H⋯O bonds that form a S (**6**) hydrogen-bonded loop. The molecules are connected in the form of dimers by C-H⋯O bonds to make a $R_{2}^{2}$(**11**) loop. The π⋯π stacking interaction between aromatic rings with inter-centroid distance range from 3.602 to 4.252 Å helps with additional stability of crystal packing. DFT calculations were performed for compound (**5**). These provided us with an understanding of the geometrical features. FMO analysis was performed to observe the reactivity pattern of the compound and also to calculate the energy difference between the HOMO and the LUMO. Hyperpolarizability studies showed that the compound showed a moderate non-linear optical response. DFT studies showed HOMO’s localization is localized over the substituted aromatic ring (right side), and the LUMO exists mainly over benzazole. This further suggests that the nucleophilic side of the molecule is the right side (according to Figure 6), while the indole side is electrophilic. A study of Hirshfeld charges helped with the analysis of charge distribution over the molecule. All the data points out the chemical reactivity of the product (**5**). Preliminary studies regarding in vitro and in silico antimicrobial activities of (**5**) against *E. coli* and *S. aureus* shows this compound can be a good nucleus for developing a further antibacterial compound.

## Figures and Tables

**Table 1:** The IR and UV data of *N*-protected alanine **4**.

ύ (cm^−1^)		λ_max_ (nm)	log ε (L.cm^−1^.M^−1^)
O–H	C=O		
3480	1720	295.5	3.2003

**Table 2:** The ^1^H-NMR signals of *N*-protected alanine **4**.

δ ppm (*J* = Hz)		
H^2^	H^3^	H^1′–4′^
5.00 (q, *J* = 7.2 Hz)	1.65 (d, *J* = 7.2 Hz)	7.85 (m)

**Table 3:** Hirshfeld charge distribution in compound (**5**) calculated at PBE0-D3BJ/def2TZVP level of theory.

Atom ^	* H.C.	Atom	* H.C.	Atom	* H.C.
O1	−0.27	O5	−0.26	C20	0.19
O2	−0.27	N6	0.00	C28	0.07
O3	−0.28	N8	−0.06	C38	0.22
O4	−0.11	C9	0.19	C39	0.15

**^** Atom numbering (in the format symbol-number) is from Figure 3; * H.C. = Hirshfeld charge.

**Table 4:** Single-crystal XRD data of **5** (CCDC No. 2059037) (Supplementary Materials; Cif file, checkcif format).

Crystallographic Data	
Chemical Formula	C_19_H_16_N_2_O_5_
*M*_r_ Temperature (K)	352.34 296 (**2**)
Crystal system, space group	Monoclinic, *P*2_1_/*n*
*a*, b, *c* (Å)	12.0628 (**10**), 9.7196 (**8**), 14.8898 (**10**)
α, β, γ° *Z*	90, 96.520 (**4**), 90 4
V (Å^3^)	1734.5 (**2**)
F(000) Wavelength (λ)	736 0.71073
Radiation type	Mo *K*α
µ (mm^−1^)	0.099
Crystal size (mm) Density (calculated)g/cm^−3^	0.30 × 0.25 × 0.23 1.349
**Data Collection**	
Absorption correction Diffractometer	Multi-scan (SADABS; Bruker, 2007) Bruker APEXII CCD diffractometer
No. of measured, independent and observed [*I* > 2σ(*I*)] reflections	14529, 4154, 2311
*R*_int_	0.032
Theta range for data collection (°)	2.062 to 27.944
Index ranges	−15 ≤ h ≤ 15, −10 ≤ k ≤ 12, −19 ≤ l ≤ 19
(sin θ/λ)_max_ (Å^−1^)	0.659
**Data Refinement**	
No. of parameters No. of reflections *R*[*F*^2^ >2σ(*F*^2^)], *wR*(*F*^2^), *S*	237 4154 0.051, 0.135, 1.01
H-atom treatment	H-atom parameters constrained
Δρ_max_, Δρ_min_ (e Å^−3^)	0.16, −0.17

**Table 5:** Selected bond lengths in angstrom and bond angles in degrees in **5**.

Bond Lengths		Bond Angles	
O1-C1	1.206 (**2**)	O1-C1-N2	124.3 (**2**)
N2-C1	1.387 (**2**)	C1-N2-C8	111.4 (**1**)
N2-C8	1.389 (**2**)	N2-C8-O2	125.1 (**2**)
O2-C8	1.201 (**2**)	C1-N2-C9	122.9 (**2**)
N2-C9	1.460 (**2**)	C8-N2-C9	124.9 (**2**)
O3-C11	1.209 (**2**)	O3-C11-N1	124.7 (**2**)
N1-C11	1.348 (**2**)	C11-N1-C12	129.7 (**2**)
N1-C12	1.396 (**2**)	O4-C18-O5	121.5(**2**)
O4-C18	1.331 (**2**)	C17-C18-O4	112.5 (**2**)
O5-C18	1.202 (**3**)	C17-C18-O5	125.9 (**2**)

**Table 6:** Hydrogen-bond geometry (Å, º) for **5**.

*D*—H⋯*A*	H⋯*A*	*D*—H	*D*⋯*A*	*<(D*—H⋯*A)°*
C9—H9⋯O2^i^	2.54	0.98	3.337 (**2**)	139
C10—H10*A*⋯O1^ii^	2.57	0.96	3.402 (**3**)	145
C19—H19*A*⋯O1^iii^	2.48	0.96	3.025 (**3**)	116
C10—H10*A*⋯O2	2.63	0.96	3.139 (**2**)	113
C13—H13⋯O3	2.24	0.93	2.857 (**2**)	123
N1—H1⋯O5	1.95	0.86	2.6562 (**19**)	139

**Table 7:** Binding site residues and binding energies of (**5**) against target enzymes.

Enzyme	PDB-ID	Binding Energy	Dissociation Constant (µM)	Active Site Residues
Dihydroorotase (*E. coli*)	2KZN	−8.05	1.25	Gly38, Ala39, Thr42, Asp40, Gly49, His50, Phe54, Pro53, Gly79, Asp80, Arg88, Lys84, Tyr170, Gln174, Gly193, Gly192, Ser194, Asp195, Asn199, Gln196, Pro222, Val224, Leu223
		−6.42	19.65	Lys226, Asp228, Lys230, Lys231, Gly233, Lys234, Ser235, Val240, Thr247, Glu251, Gln254, Phe255, Asn258
DNA gyrase (*E. coli*)	2EG7	−7.47	3.40	His18, Arg20, Kcx102, Asn44, Tyr79, Tyr104, Pro105, Thr110, Asn107, His139, Thr143, His177, Leu222, Asp250, His254, Ala252, Ala266
		−7.20	6.31	Asn44, Tyr79, Pro105, Tyr104, Asn107, Asn111, Thr110, His114, Gly115, Val116, Leu222, His139
Tyrosyl-tRNA synthetase (*S. aureus*)	1JIJ	−8.47	0.615	Gly38, Ala39, Thr42, Asp40, Gly49, Pro53, His50, Phe54, Gly79, Asp80, Lys84, Gln174, Arg88, Tyr170, Gly193, Ser194, Gly192, Asp195
		−7.86	1.72	Arg58, Asn109, Phe271, Thr272, Phe273, Glu302, Val303, Lys305, Phe306, Gly309, Glu310, Asp311
DNA gyrase (*S. aureus*)	3G7B	−6.77	10.79	Lys78, Thr80, Asp81, Asn82, Tyr141, His143, Ile148, Lys170, Thr171, Gly172, Val174, Arg176
		−6.62	14.11	Gln66, Ile67, Glu68, Thr80, Asp81, Asn82, His143, Lys170, Thr171, Gly172, Val174, Gln210, Thr212, Ser226

## Data Availability

Samples of the compounds are available from the authors.

## Supplementary Materials

The following supporting information can be downloaded at: https://www.mdpi.com/article/10.3390/molecules28114375/s1, supporting the formation file. Compound characterization data (1HNMR **2**, LR EIMS **2**, 1HNMR 4, IR 5, 1HNMR 5, HMBC 5, LREIMS 5, ESI MS 5), Cif file, checkcif.

## Sample Availability

Sample of the compounds **5** is available from the authors.
