# Supplementary material for: Synthesis, Crystal Structure, DFT Calculations, Hirshfeld Surface Analysis and In Silico Drug-Target Profiling of (R)-2-(2-(1,3-Dioxoisoindolin-2-yl)propanamido)benzoic Acid Methyl Ester
Source: Molecules. 2023 May 26;28(11):4375. doi: 10.3390/molecules28114375 (PMC10254292; doi:10.3390/molecules28114375)

checkCIF/PLATON report

Structure factors have been supplied for datablock(s) lr_ii_797_0m

THIS REPORT IS FOR GUIDANCE ONLY. IF USED AS PART OF A REVIEW PROCEDURE FOR PUBLICATION, IT SHOULD NOT REPLACE THE EXPERTISE OF AN EXPERIENCED CRYSTALLOGRAPHIC REFEREE.

No syntax errors found.   CIF dictionary   Interpreting this report

Datablock: lr_ii_797_0m

```
Bond precision:  C-C = 0.0029 A          Wavelength=0.71073

Cell:            a=12.0628(10)    b=9.7196(8)       c=14.8898(10)
                 alpha=90         beta=96.520(4)    gamma=90
Temperature:     296 K

                 Calculated                Reported
Volume           1734.5(2)                 1734.5(2)
Space group      P 21/n                    P 21/n
Hall group       -P 2yn                    -P 2yn
Moiety formula   C19 H16 N2 O5             C19 H16 N2 O5
Sum formula      C19 H16 N2 O5             C19 H16 N2 O5
Mr               352.34                    352.34
Dx,g cm-3        1.349                     1.349
Z                4                         4
Mu (mm-1)        0.099                     0.099
F000             736.0                     736.0
F000'            736.39
h,k,lmax         15,12,19                  15,12,19
Nref             4163                      4154
Tmin,Tmax        0.971,0.977               0.980,0.985
Tmin'            0.971

Correction method= # Reported T Limits: Tmin=0.980 Tmax=0.985
AbsCorr = MULTI-SCAN

Data completeness= 0.998          Theta(max)= 27.944

R(reflections)= 0.0505( 2311)     wR2(reflections)= 0.1352( 4154)

S = 1.012                   Npar= 237
```

The following ALERTS were generated. Each ALERT has the format
test-name_ALERT_alert-type_alert-level.
Click on the hyperlinks for more details of the test.

Alert level C

```
PLAT334_ALERT_2_C Small Aver. Benzene C-C Dist C2       -C7             1.37 Ang.
PLAT480_ALERT_4_C Long H...A H-Bond Reported H10A     ..O2       .       2.63 Ang.
PLAT906_ALERT_3_C Large K Value in the Analysis of Variance ......      4.033 Check
```

Alert level G

```
PLAT007_ALERT_5_G Number of Unrefined Donor-H Atoms ..............          1 Report
PLAT793_ALERT_4_G Model has Chirality at C9          (Centro SPGR)          S Verify
PLAT912_ALERT_4_G Missing # of FCF Reflections Above STh/L=  0.600         10 Note
PLAT941_ALERT_3_G Average HKL Measurement Multiplicity ...........        3.5 Low
PLAT978_ALERT_2_G Number C-C Bonds with Positive Residual Density.          1 Info
```

```
   0 ALERT level A = Most likely a serious problem - resolve or explain
   0 ALERT level B = A potentially serious problem, consider carefully
   3 ALERT level C = Check. Ensure it is not caused by an omission or oversight
   5 ALERT level G = General information/check it is not something unexpected

   0 ALERT type 1 CIF construction/syntax error, inconsistent or missing data
   2 ALERT type 2 Indicator that the structure model may be wrong or deficient
   2 ALERT type 3 Indicator that the structure quality may be low
   3 ALERT type 4 Improvement, methodology, query or suggestion
   1 ALERT type 5 Informative message, check
```

It is advisable to attempt to resolve as many as possible of the alerts in all categories. Often the minor alerts point to easily fixed oversights, errors and omissions in your CIF or refinement strategy, so attention to these fine details can be worthwhile. In order to resolve some of the more serious problems it may be necessary to carry out additional measurements or structure refinements. However, the purpose of your study may justify the reported deviations and the more serious of these should normally be commented upon in the discussion or experimental section of a paper or in the "special_details" fields of the CIF. checkCIF was carefully designed to identify outliers and unusual parameters, but every test has its limitations and alerts that are not important in a particular case may appear. Conversely, the absence of alerts does not guarantee there are no aspects of the results needing attention. It is up to the individual to critically assess their own results and, if necessary, seek expert advice.

Publication of your CIF in IUCr journals

A basic structural check has been run on your CIF. These basic checks will be run on all CIFs submitted for publication in IUCr journals (*Acta Crystallographica*, *Journal of Applied Crystallography*, *Journal of Synchrotron Radiation*); however, if you intend to submit to *Acta Crystallographica Section C* or *E* or *IUCrData*, you should make sure that full publication checks are run on the final version of your CIF prior to submission.

Publication of your CIF in other journals

Please refer to the *Notes for Authors* of the relevant journal for any special instructions relating to CIF submission.

PLATON version of 05/12/2020; check.def file version of 05/12/2020

Datablock lr_ii_797_0m - ellipsoid plot

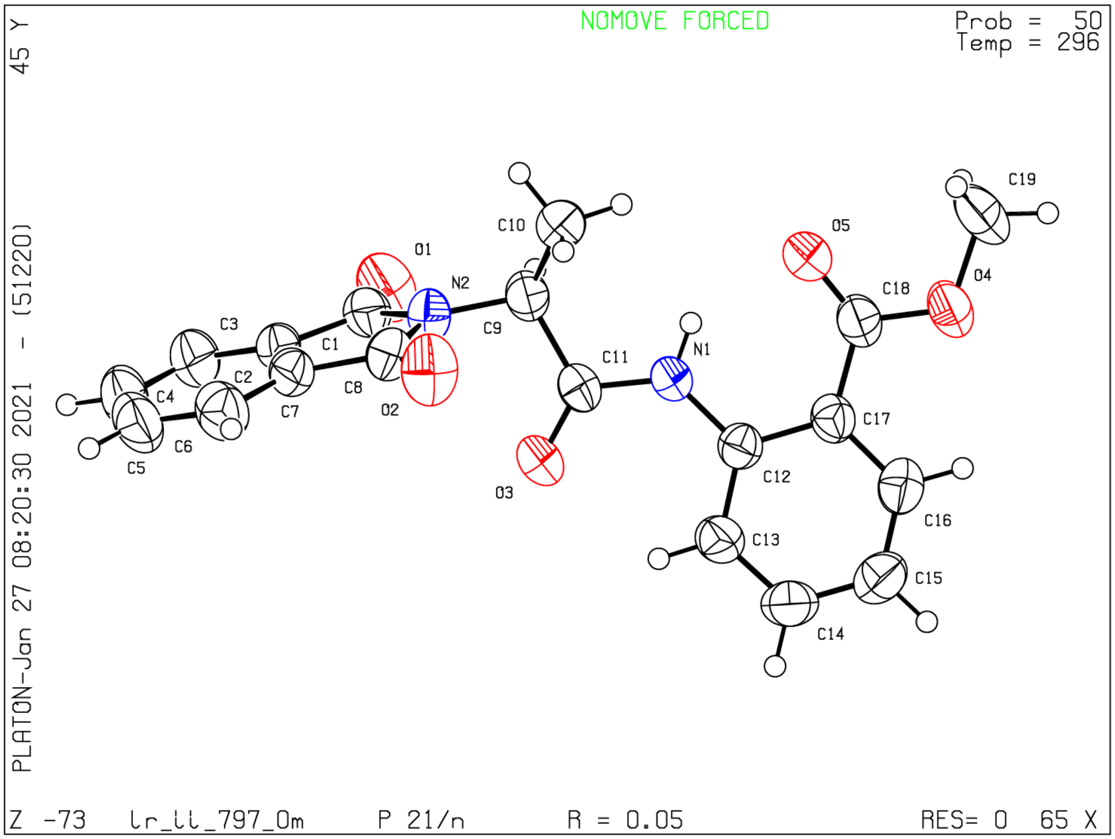

Supplement: Supplementary file 1 [file molecules-28-04375-s001.zip › checkcif report of lr_ii_797_0m.pdf]
