# Supplementary material for: Synthesis, Crystal Structure, DFT Calculations, Hirshfeld Surface Analysis and In Silico Drug-Target Profiling of (R)-2-(2-(1,3-Dioxoisoindolin-2-yl)propanamido)benzoic Acid Methyl Ester
Source: Molecules. 2023 May 26;28(11):4375. doi: 10.3390/molecules28114375 (PMC10254292; doi:10.3390/molecules28114375)

^{1}HNMR **2**

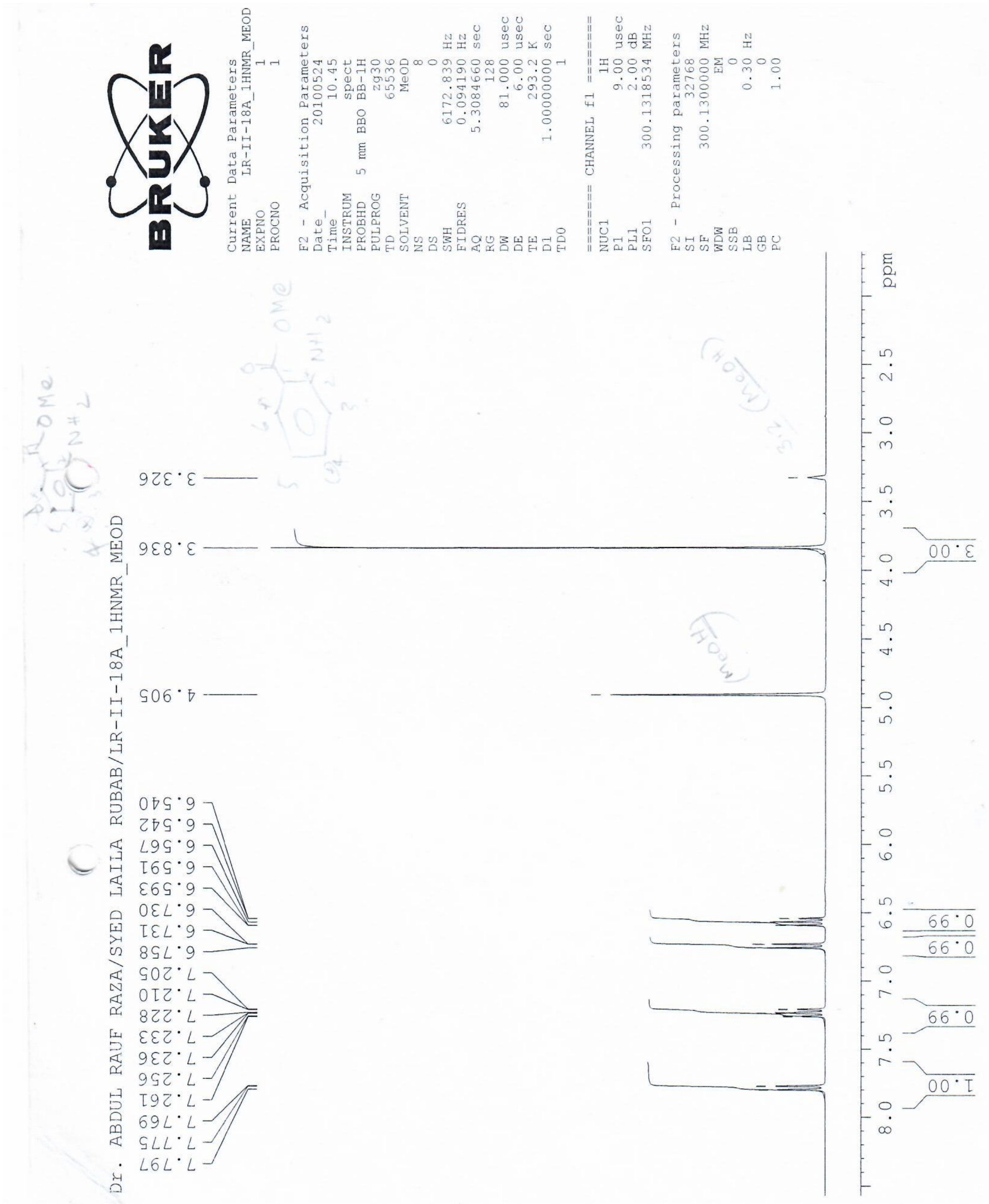

Dr. ABDUL RAUF RAZA/SYED LAILA RUBAB/LR-II-18A_1HNMR_MEOD
3.326
3.836
4.905
6.540
6.542
6.567
6.591
6.593
6.730
6.731
6.758
7.205
7.210
7.228
7.233
7.236
7.256
7.261
7.769
7.775
7.797
BRUKER
Current Data Parameters
NAME LR-II-18A_1HNMR_MEOD
EXPNO 1
PROCNO 1
F2 - Acquisition Parameters
Date_ 20100524
Time 10.45
INSTRUM spect
PROBHD 5 mm BBO BB-1H
PULPROG zg30
TD 65536
SOLVENT MeOD
NS 8
DS 0
SWH 6172.839 Hz
FIDRES 0.094190 Hz
AQ 5.3084660 sec
RG 128
DW 81.000 usec
DE 6.00 usec
TE 293.2 K
D1 1.00000000 sec
TD0 1
======== CHANNEL f1 ========
NUC1 1H
P1 9.00 usec
PL1 2.00 dB
SFO1 300.1318534 MHz
F2 - Processing parameters
SI 32768
SF 300.1300000 MHz
WDW EM
SSB 0
LB 0.30 Hz
GB 0
PC 1.00
3.00
0.99
0.99
0.99
1.00
ppm

Dr. ABDUL RAUF RAZA/SYED LAILA RUBAB/LR-II-18A_1HNMR_MEOD

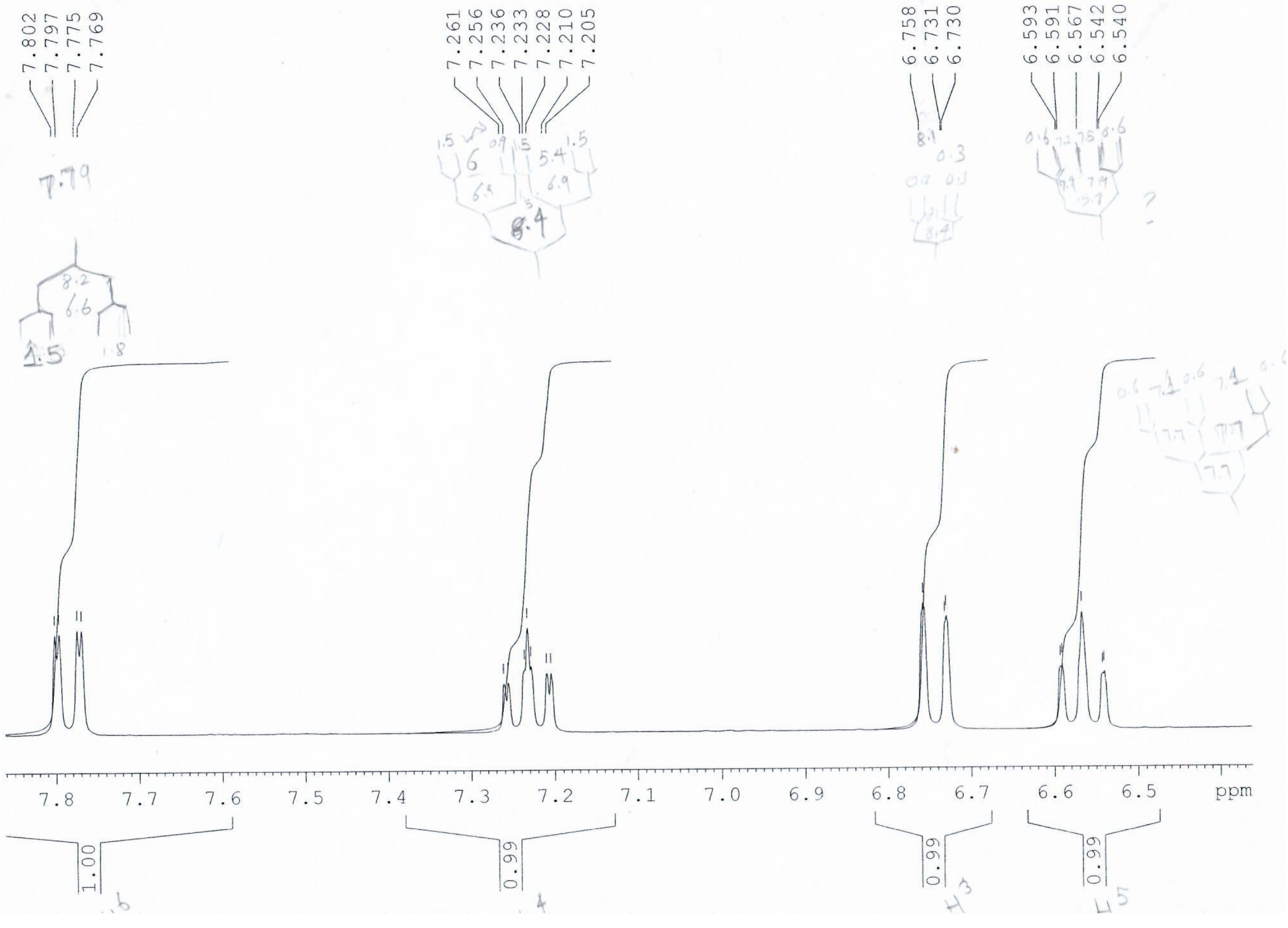

LR EIMS **2**

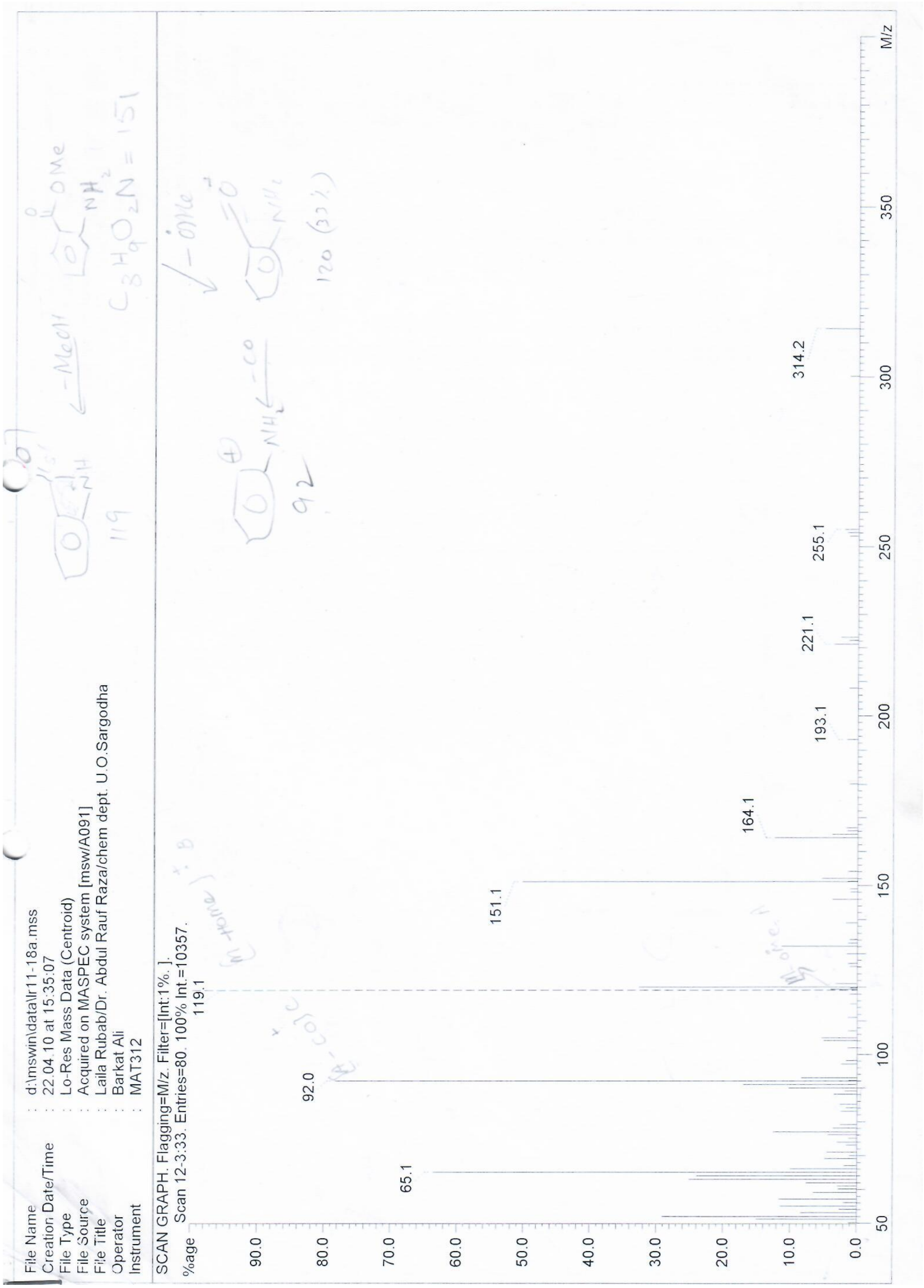

File Name : d:\mswin\data\lr11-18a.mss
Creation Date/Time : 22.04.10 at 15:35:07
File Type : Lo-Res Mass Data (Centroid)
File Source : Acquired on MASPEC system [msw/A091]
File Title : Laila Rubab/Dr. Abdul Rauf Raza/chem dept. U.O.Sargodha
Operator : Barkat Ali
Instrument : MAT312
SCAN GRAPH. Flagging=M/z. Filter=[Int:1%.].
Scan 12-3:33. Entries=80. 100% Int.=10357.
%age
M/z
65.1
92.0
119.1
151.1
164.1
193.1
221.1
255.1
314.2

^{1}HNMR **4**

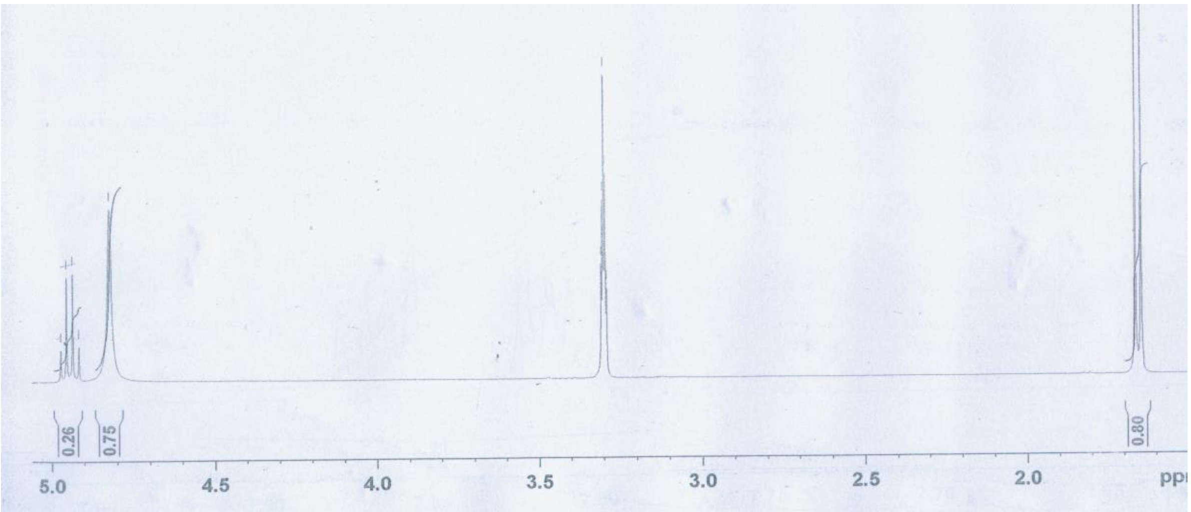

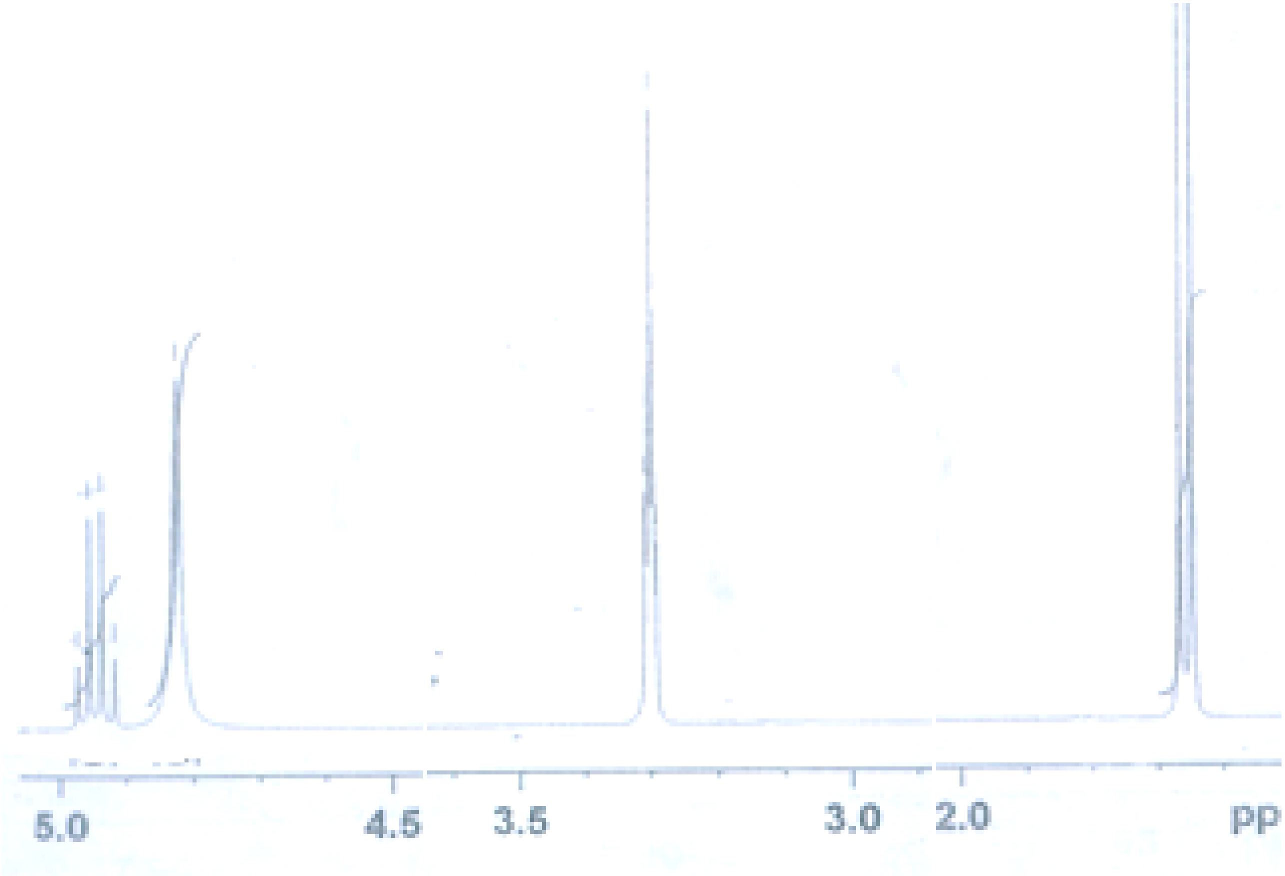

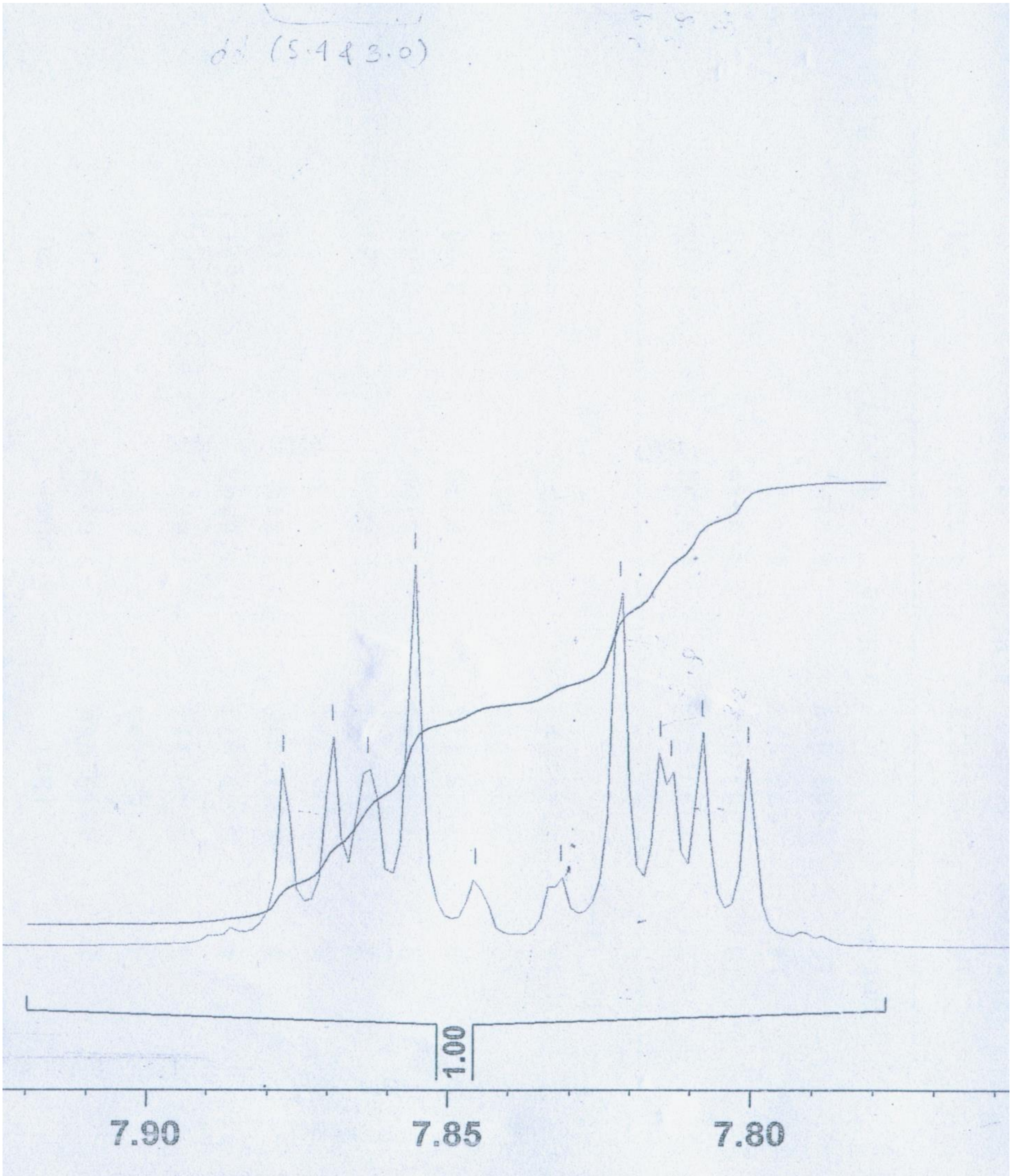

dd (5.4 & 3.0)
1.00
7.90
7.85
7.80

IR 5

SHIMADZU

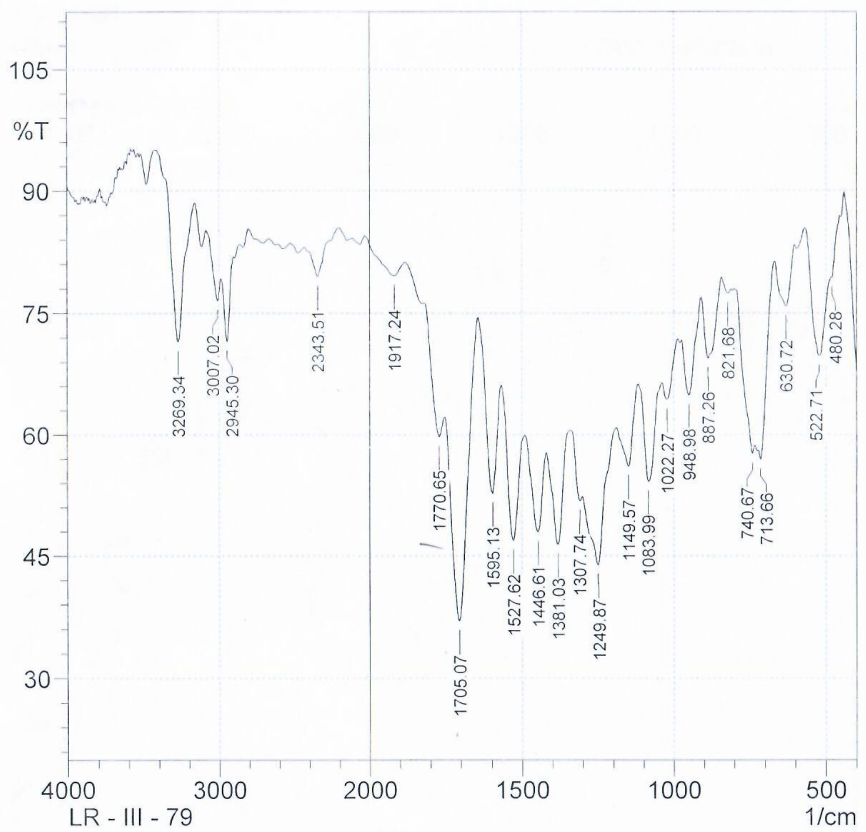

	Peak	Height	Corr. He	Base (H)	Base (L)	Area	Corr. Ar
1	480.28	20.69	0.64	482.2	455.2	2.13	0.04
2	522.71	30.22	12.28	569	484.13	10.29	3.07
3	630.72	24.13	6.66	667.37	603.72	6.76	1.41
4	713.66	43.08	4.62	721.38	669.3	8.79	0.62
5	740.67	42.36	3.51	792.74	732.95	11.29	1.01
6	821.68	22.58	0.99	840.96	812.03	3.13	0.1
7	887.26	30.54	8.23	908.47	842.89	8.93	1.92
8	948.98	35.11	8.88	974.05	910.4	10.05	1.86
9	1022.27	35.63	3.7	1037.7	987.55	8.68	0.66
10	1083.99	45.82	12.15	1116.78	1039.63	16.76	3.01
11	1149.57	43.92	7.79	1188.15	1118.71	15.58	1.93
12	1249.87	56.14	12.35	1300.02	1190.08	32.23	4.96
13	1307.74	48.21	1.94	1334.74	1301.95	8.57	0.33
14	1381.03	53.61	12.7	1417.68	1342.46	20.69	3.53
15	1446.61	52.08	10.72	1489.05	1419.61	19.07	3.08
16	1527.62	53.12	16.07	1566.2	1490.97	19.69	4.53
17	1595.13	47.31	16.49	1641.42	1568.13	15.51	4.18
18	1705.07	63.01	30.49	1751.36	1643.35	30.98	13.02
19	1770.65	40.28	6.28	1816.94	1753.29	11.83	1.43
20	1917.24	20.43	2.39	2031.04	1884.45	12.96	0.97
21	2343.51	20.53	3.64	2399.45	2262.5	12.06	1.16
22	2945.3	28.51	9	2983.88	2900.94	9.69	1.92
23	3007.02	23.49	4.01	3082.25	2985.81	9.15	0.9
24	3269.34	28.54	19.87	3408.22	3161.33	19.01	9.67

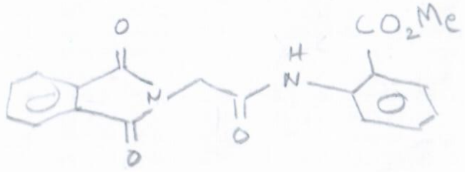

Comment;
LR - II[illegible]- 79

Date/Time; 9/14/2012 9:06:15 AM
No. of Scans;
Resolution;
Apodization;

^{1}HNMR **5**

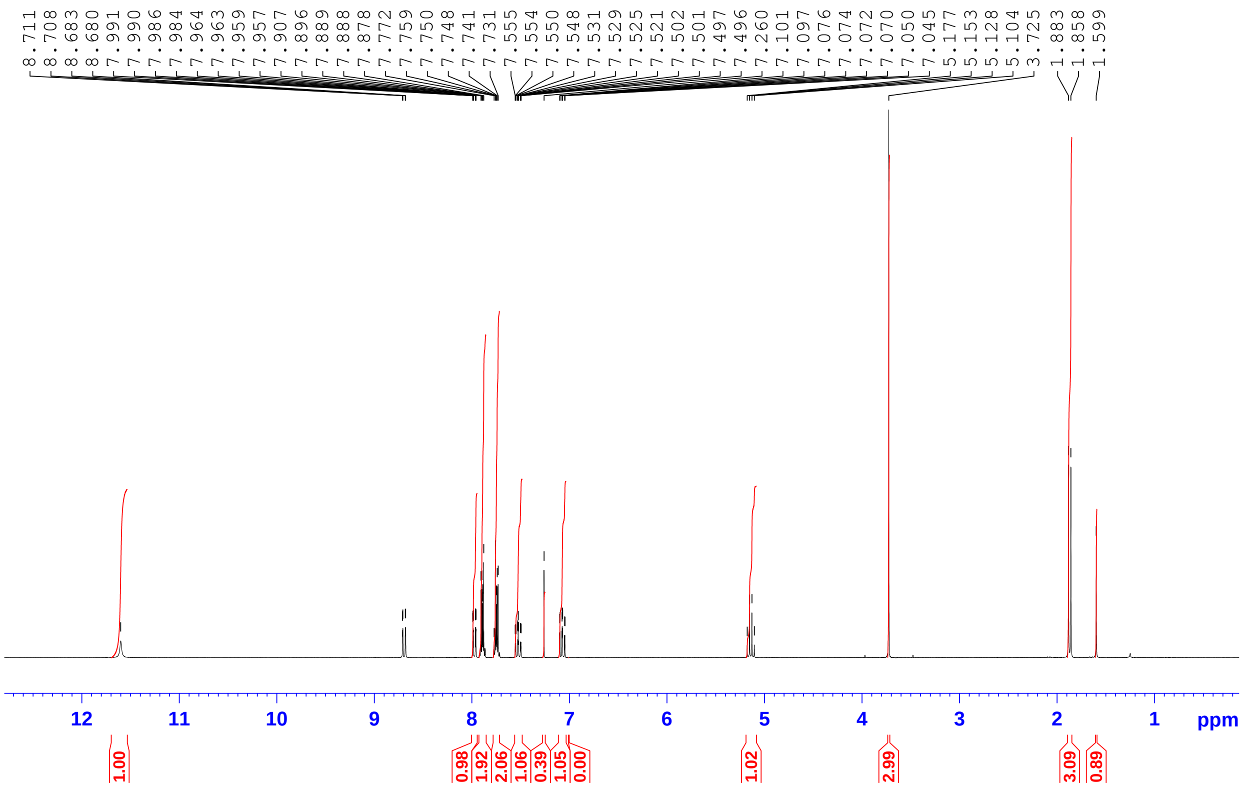

8.711
8.708
8.683
8.680
7.991
7.990
7.986
7.984
7.964
7.963
7.959
7.957
7.907
7.896
7.889
7.888
7.878
7.772
7.759
7.750
7.748
7.741
7.731
7.555
7.554
7.550
7.548
7.531
7.529
7.525
7.521
7.502
7.501
7.497
7.496
7.260
7.101
7.097
7.076
7.074
7.072
7.070
7.050
7.045
5.177
5.153
5.128
5.104
3.725
1.883
1.858
1.599
12
11
10
9
8
7
6
5
4
3
2
1
ppm
1.00
0.98
1.92
2.06
1.06
0.39
1.05
0.00
1.02
2.99
3.09
0.89

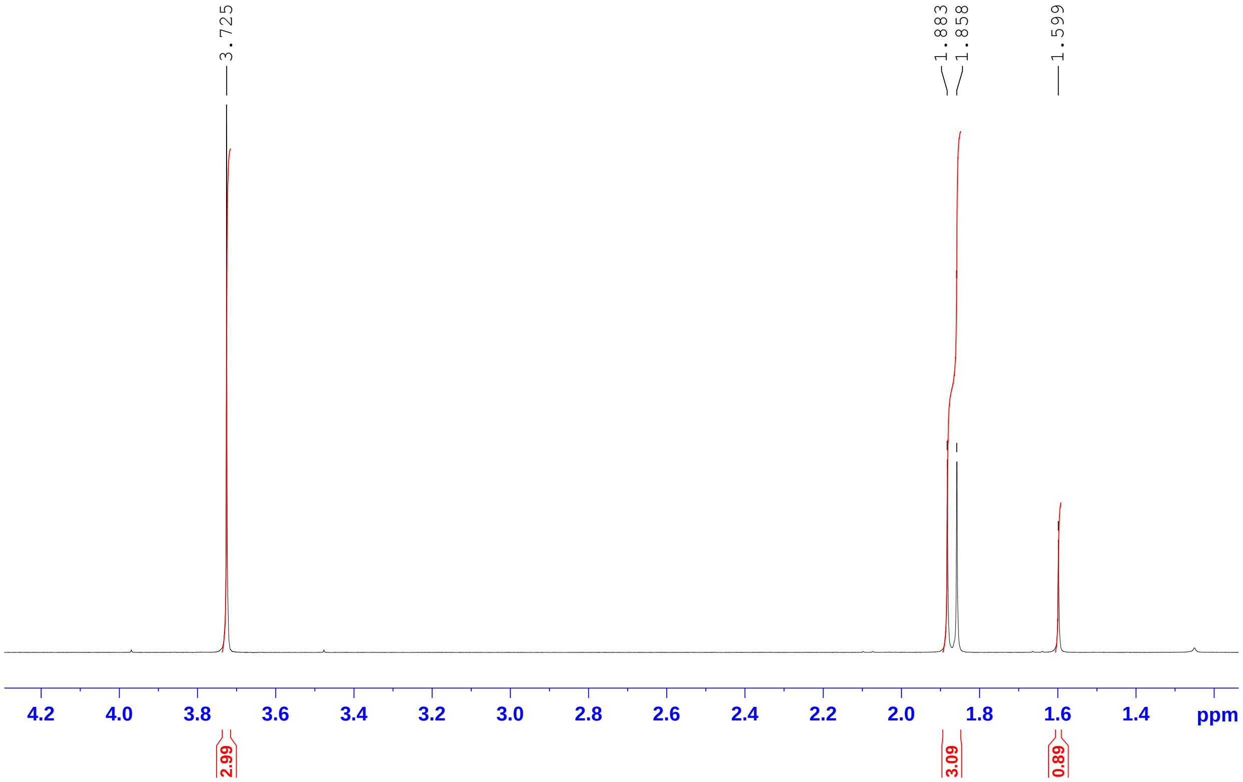
3.725
1.883
1.858
1.599
4.2 4.0 3.8 3.6 3.4 3.2 3.0 2.8 2.6 2.4 2.2 2.0 1.8 1.6 1.4 ppm
2.99
3.09
0.89

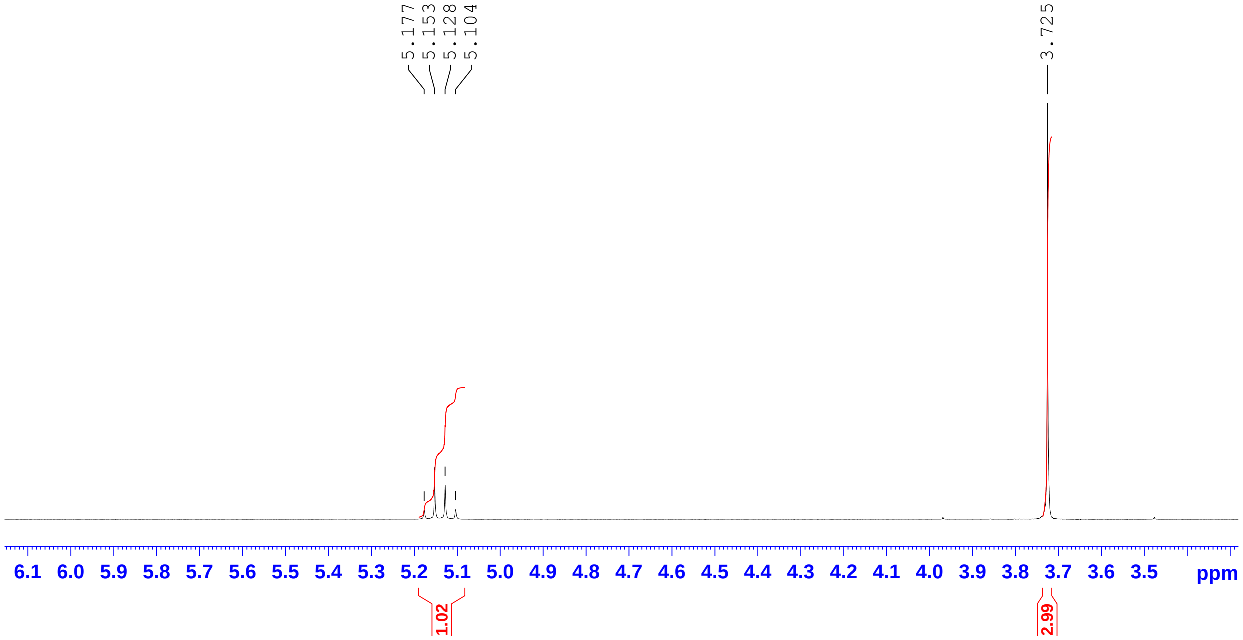
5.177
5.153
5.128
5.104
3.725
6.1 6.0 5.9 5.8 5.7 5.6 5.5 5.4 5.3 5.2 5.1 5.0 4.9 4.8 4.7 4.6 4.5 4.4 4.3 4.2 4.1 4.0 3.9 3.8 3.7 3.6 3.5 ppm
1.02
2.99

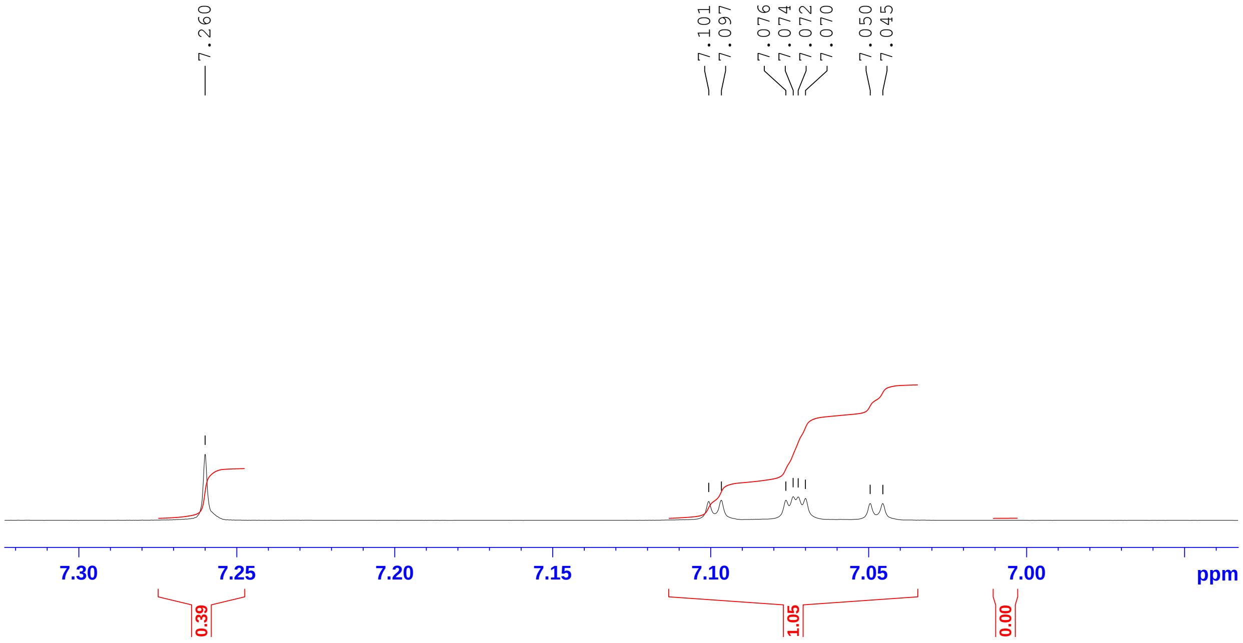
7.260
7.101
7.097
7.076
7.074
7.072
7.070
7.050
7.045
7.30
7.25
7.20
7.15
7.10
7.05
7.00
ppm
0.39
1.05
0.00

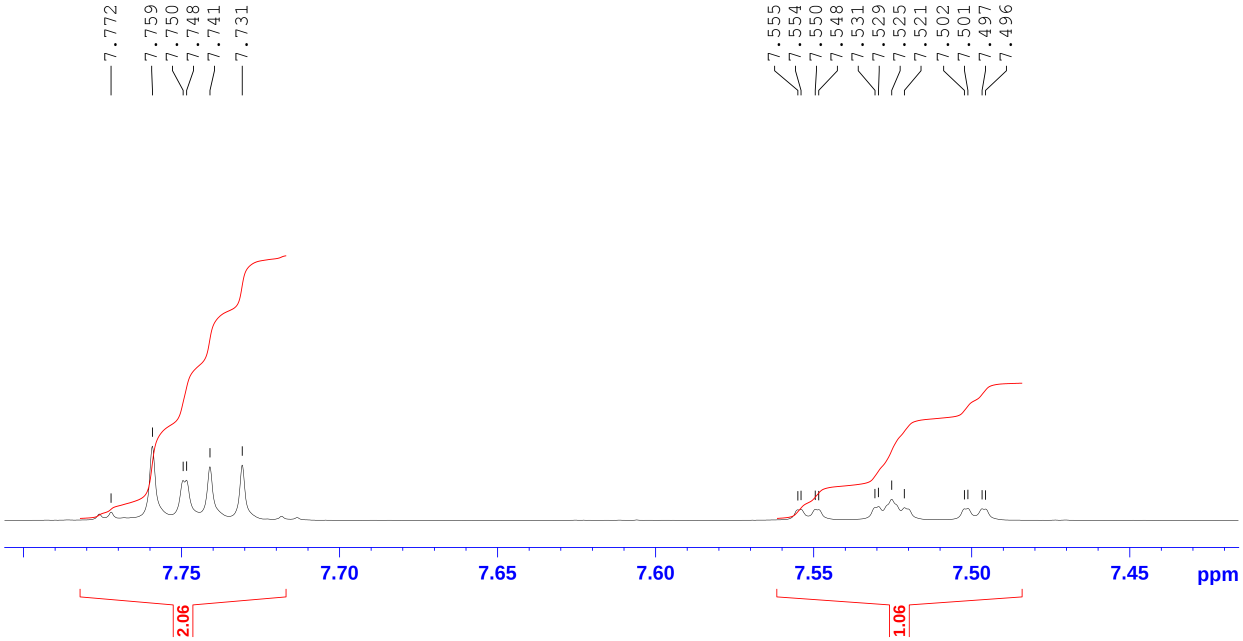
7.772
7.759
7.750
7.748
7.741
7.731
7.555
7.554
7.550
7.548
7.531
7.529
7.525
7.521
7.502
7.501
7.497
7.496
7.75
7.70
7.65
7.60
7.55
7.50
7.45
ppm
2.06
1.06

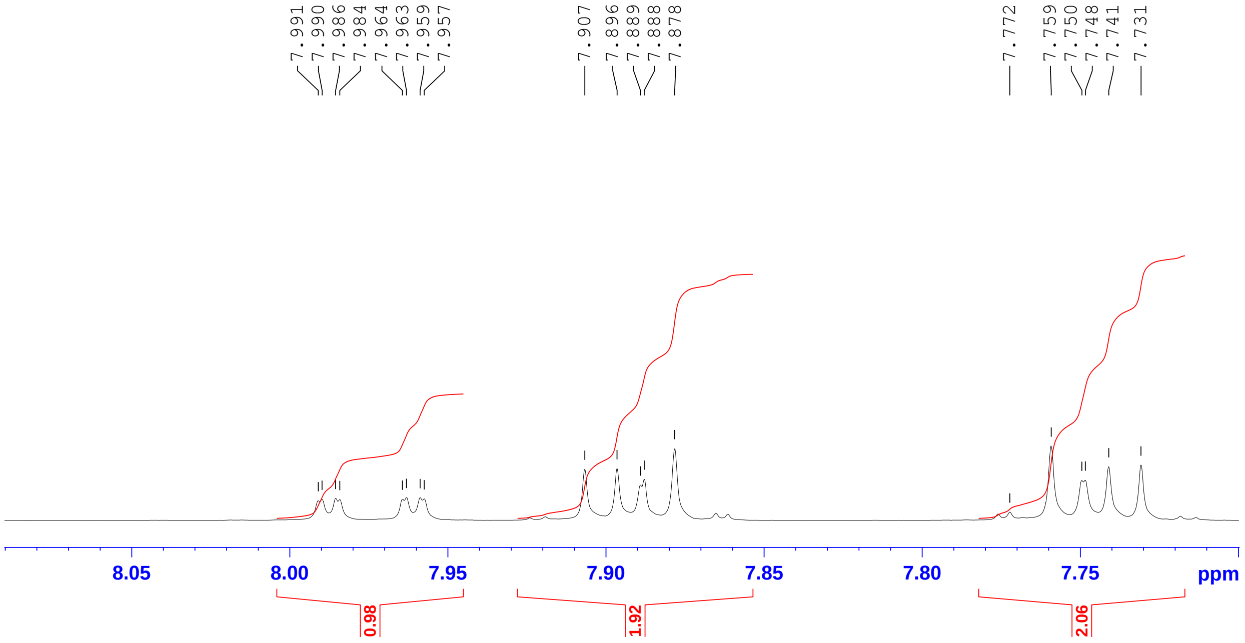
7.991
7.990
7.986
7.984
7.964
7.963
7.959
7.957
7.907
7.896
7.889
7.888
7.878
7.772
7.759
7.750
7.748
7.741
7.731
8.05
8.00
7.95
7.90
7.85
7.80
7.75
ppm
0.98
1.92
2.06

^{13}CNMR **5**

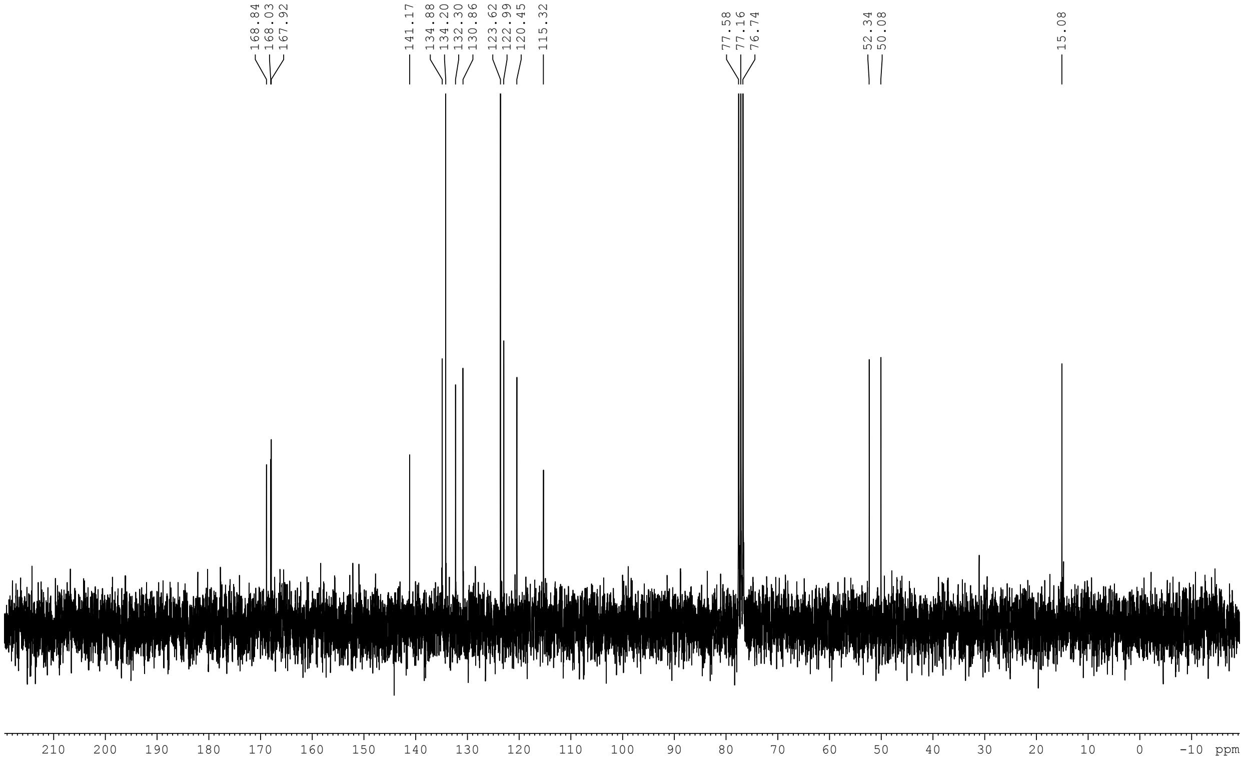
168.84
168.03
167.92
141.17
134.88
134.20
132.30
130.86
123.62
122.99
120.45
115.32
77.58
77.16
76.74
52.34
50.08
15.08
210
200
190
180
170
160
150
140
130
120
110
100
90
80
70
60
50
40
30
20
10
0
-10
ppm

HMBC 5

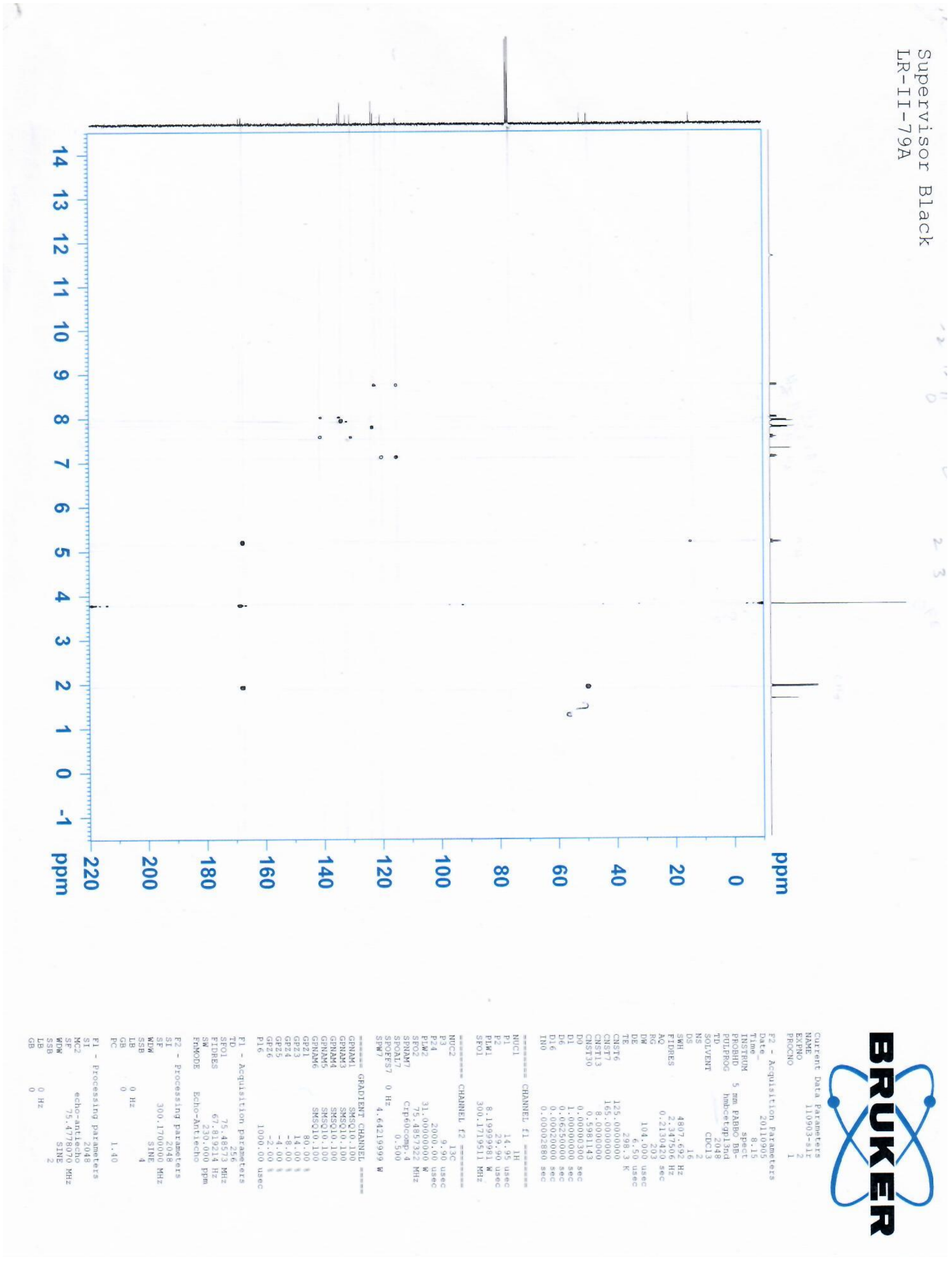

LREIMS **5**

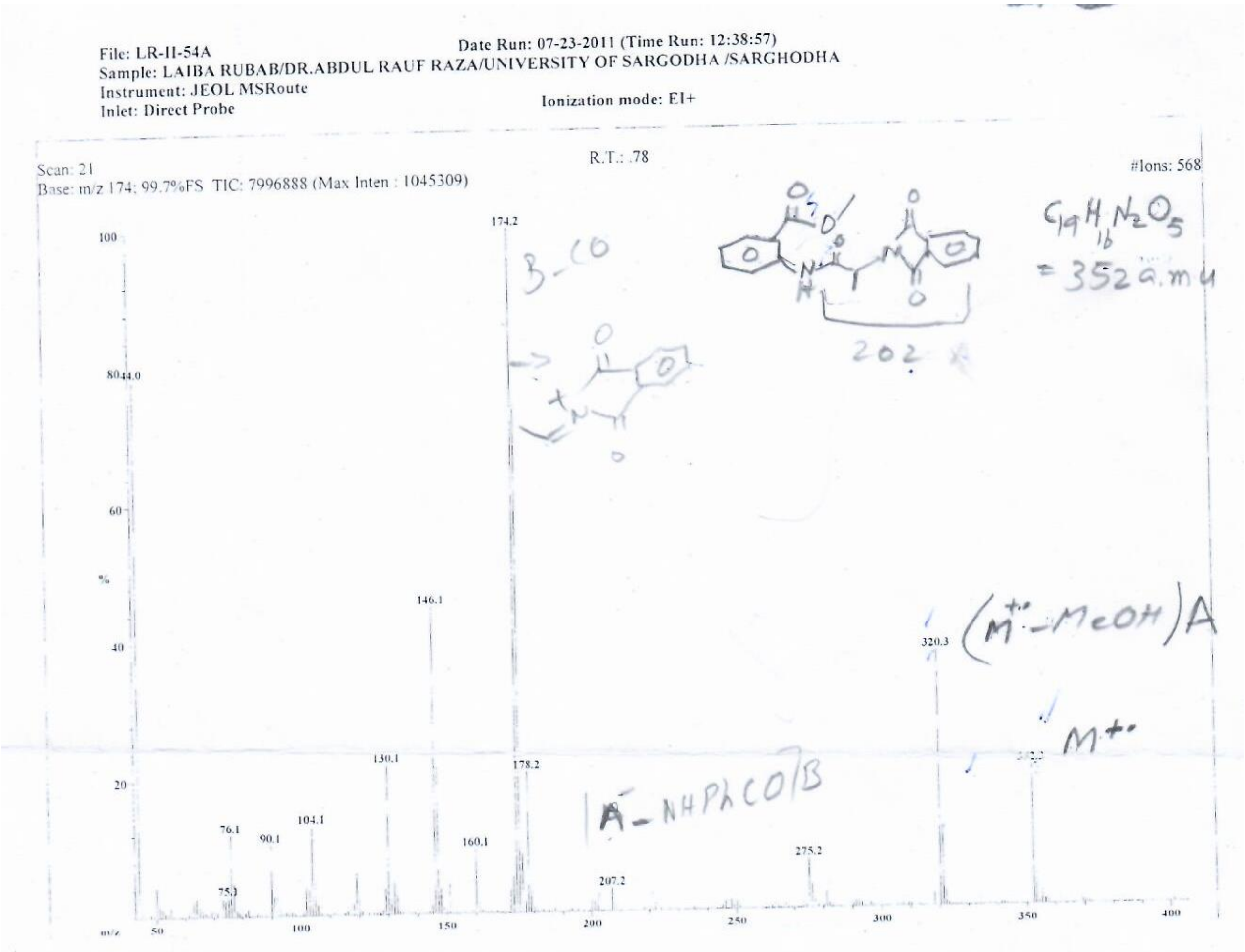
File: LR-II-54A
Date Run: 07-23-2011 (Time Run: 12:38:57)
Sample: LAIBA RUBAB/DR.ABDUL RAUF RAZA/UNIVERSITY OF SARGODHA /SARGHODHA
Instrument: JEOL MSRoute
Inlet: Direct Probe
Ionization mode: EI+
Scan: 21
Base: m/z 174; 99.7%FS TIC: 7996888 (Max Inten : 1045309)
R.T.: .78
#Ions: 568
174.2
146.1
130.1
178.2
104.1
76.1
90.1
160.1
75.1
207.2
275.2
320.3
m/z
%

ESI MS 5

MS Data from Orbitrap

Tracking No: BMSF-2012-8
Samples Submitted By: Syeda Laila Rubub
Date Run: 31/01/12
Operator: Chowdhury Sarowar
Report Prepared By: Leanne Stephenson

Sample: LR-II

Full Spectrum

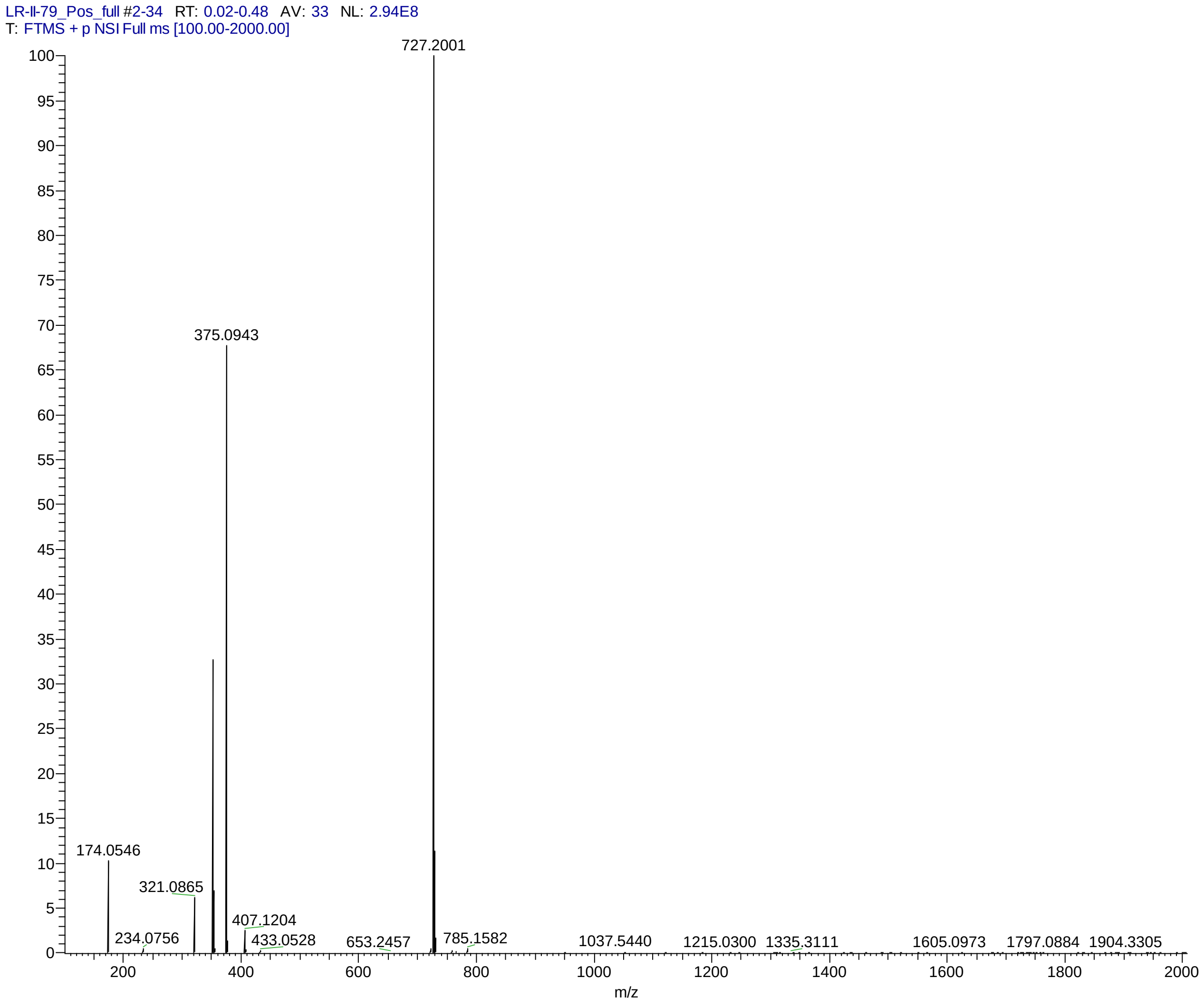

Zoomed Spectrum

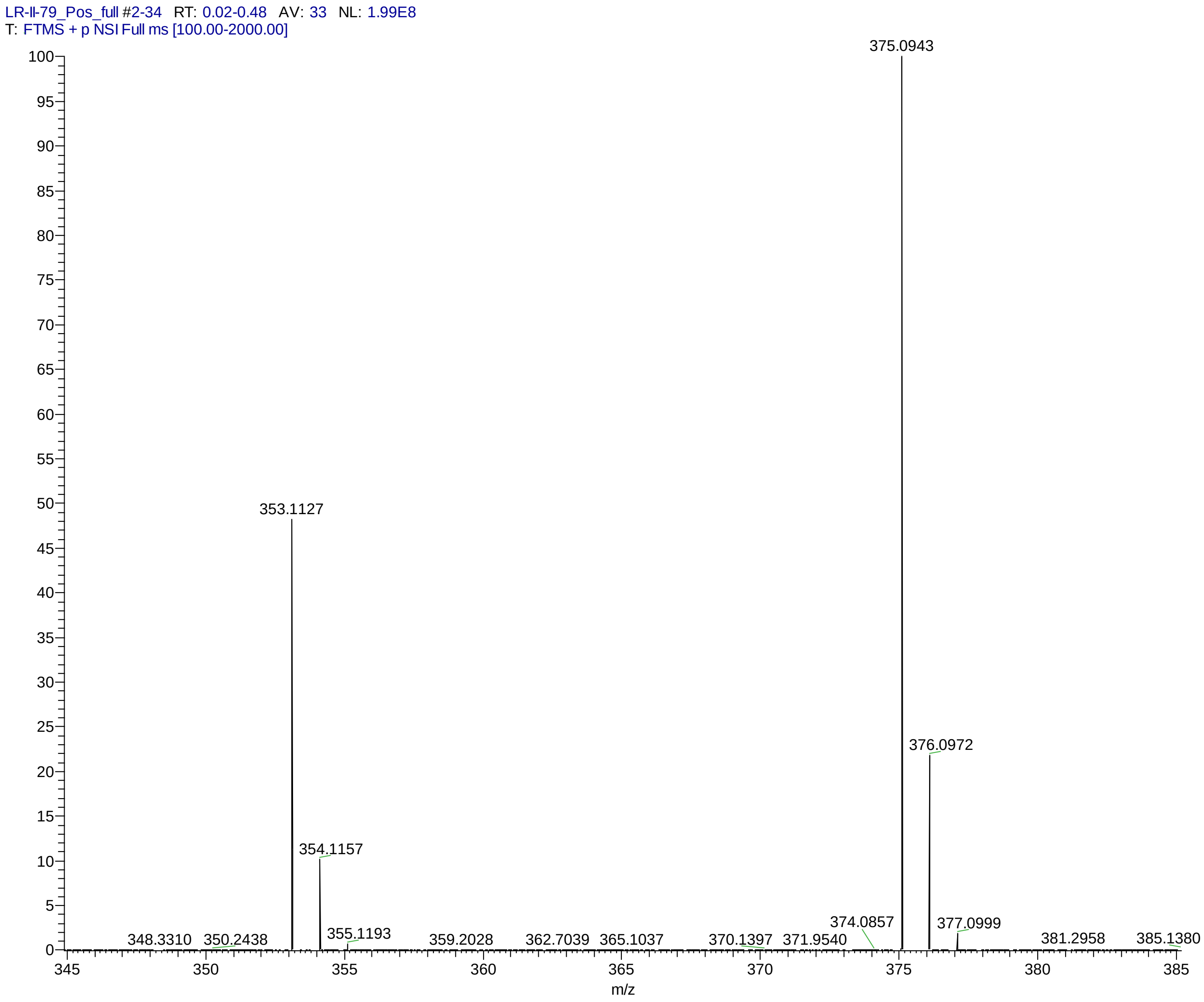

Supplement: Supplementary file 1 [file molecules-28-04375-s001.zip › Supplementary data.pdf]
